# Central Pattern Generator for Locomotion: Anatomical, Physiological, and Pathophysiological Considerations

**DOI:** 10.3389/fneur.2012.00183

**Published:** 2013-02-08

**Authors:** Pierre A. Guertin

**Affiliations:** ^1^Department of Psychiatry and Neurosciences, Laval UniversityQuebec City, QC, Canada; ^2^Laval University Medical Center (CHU de Quebec)Quebec City, QC, Canada

**Keywords:** spinal networks, central pattern generator for locomotion, rhythmic stereotyped patterns, restless leg syndrome, alternating leg movement activation, Uner Tan syndrome

## Abstract

This article provides a perspective on major innovations over the past century in research on the spinal cord and, specifically, on specialized spinal circuits involved in the control of rhythmic locomotor pattern generation and modulation. Pioneers such as Charles Sherrington and Thomas Graham Brown have conducted experiments in the early twentieth century that changed our views of the neural control of locomotion. Their seminal work supported subsequently by several decades of evidence has led to the conclusion that walking, flying, and swimming are largely controlled by a network of spinal neurons generally referred to as the central pattern generator (CPG) for locomotion. It has been subsequently demonstrated across all vertebrate species examined, from lampreys to humans, that this CPG is capable, under some conditions, to self-produce, even in absence of descending or peripheral inputs, basic rhythmic, and coordinated locomotor movements. Recent evidence suggests, in turn, that plasticity changes of some CPG elements may contribute to the development of specific pathophysiological conditions associated with impaired locomotion or spontaneous locomotor-like movements. This article constitutes a comprehensive review summarizing key findings on the CPG as well as on its potential role in Restless Leg Syndrome, Periodic Leg Movement, and Alternating Leg Muscle Activation. Special attention will be paid to the role of the CPG in a recently identified, and uniquely different neurological disorder, called the Uner Tan Syndrome.

## Gross Anatomy of the Spinal Cord

The Central Pattern Generator (CPG) for locomotion could hardly be introduced properly without describing first the spinal cord itself as well as the supraspinal and peripheral systems associated with its physiological basis.

The spinal cord constitutes the most caudally located structure of the central nervous system (CNS). It is essentially a long and relatively thin neural structure that extends from the base of the skull (i.e., the medulla) to the first lumbar vertebra. It is contained within the vertebral column but does not extend the entire length of that protective bone structure. In humans, it elliptical structure varies in length between 43 and 45 cm comprising 31 different segments (species-specific variations exist) – 8 cervical, 12 thoracic, 5 lumbar, 5 sacral, and 1 coccygeal segments. Every segment is associated with a pair (right and left) of spinal nerves. The spinal nerves comprise the sensory nerve roots (composed of 6–8 rootlets), which enter the spinal cord at each level, and the motor roots (also with 6–8 rootlets), which emerge from the cord at each level. In rostral parts, the spinal nerves exit directly from (just above from C1 to C7 or below from C8 and lower) the vertebra associated numerically with the corresponding spinal cord segment. However, in caudal parts of the spinal cord, the spinal nerves travel further down the column before exiting (Netter, 2006).

In transverse sections, the spinal cord displays white and the gray matter tissues. Peripherally located, the former contains white matter tracts (ascending and descending myelinated fibers) containing both sensory and motor axons. Centrally located, the latter is characterized by its butterfly-shape that contains gray matter cells (unmyelinated) and, specifically, simple as well as more complex spinal circuits (see sections on reflexes and networks). In the center, there is a central canal that contains cerebrospinal fluids traveling up to the ventricles located in the brain. Peripherally, we find meninges that are layers of tissue surrounding the spinal cord for its protection – the dura, the arachnoid, and the pia. The latter is relatively thin and tightly associated with the surface of the spinal cord. The spinal cord is supplied by a vast system of blood vessels comprising mainly the anterior spinal artery, the bilateral sulcal branches, the bilateral posterior spinal arteries, the pial arterial plexus as well as the anterior/posterior spinal veins, the anterior/posterior sulcal veins, and the pial venous plexus (Kandel et al., 2000).

As mentioned above, each spinal cord segment is associated with a pair of nerves that innervates different skin areas and sets of muscles. In brief, cervical segments provide innervations to and from muscles involved in respiration, head, neck, and arm movements. Thoracic segments provide motor control of the finger, chest, back, and abdominal muscles whereas the lumbar and sacral segments are generally associated with the control of muscles involved in locomotion, micturition, bowel, and reproductive functions (for further details, see Guertin, 2013).

## Spinal Cord White Matter

Several tracts of fibers located in the white matter carry information between brain structures and spinal cord structures (e.g., motoneurons, simple reflexes, CPG, etc.). Ascending white matter tracts are mainly located dorsally and laterally whereas descending ones are mainly located ventrolaterally and ventrally. The ascending tracts convey sensory signals associated with the sense of touch, pressure, proprioception, and vibration via relatively large myelinated fibers (specifically via the gracile fasciculus or the cuneate fasciculus to different areas of the brain). These tracts travel contralaterally (decussation) in the medulla prior to being redirected (signals) toward the thalamus and eventually the sensory cortex (Patestas and Gartner, 2006). In contrast, the lateral or so-called anterolateral columns convey information about pain and thermal sensation and decussate instead at the spinal cord level (close to the entry of the corresponding primary afferents) prior to ascend to the brain via a number of tracts. It is beyond the scope of this review to describe them all (e.g., Lissauer’s tract, dorsal spinal tract, ventral spinal tract, spinocerebellar tract, spino-thalamic tract, etc., for further details, see Guertin, 2013).

The descending motor system is also divided in multiple tracts essentially composing the so-called pyramidal and extrapyramidal tracts. The corticospinal tracts, also called the pyramidal tracts, contain about one million axons on each side that are involved essentially in skilled movements. In contrast, the extrapyramidal tracts are mainly involved in the control of complex behaviors such as postural control and locomotion. The pyramidal tract originates from the cerebral cortex and from some brainstem motor nuclei. It constitutes the most direct descending motor pathway (e.g., some monosynaptic connections) between the motor cortex (Brodmann’s areas 1, 2, 3, 4, and 6) and the final common motor pathway, namely motoneurons in the spinal cord located at all segmental levels. Although often referred to as upper motor neurons, axons composing the pyramidal tracks have their cell bodies in the motor cortex and should accordingly and more appropriately be called corticospinal neurons. About¸ 80–90% of these corticospinal axons are known to decussate to the contralateral side at the pyramid level in the medulla oblongata. From there, they constitute the lateral corticospinal tract that sends input to spinal motoneurons in the ventral horn area. The remaining 10–20% descends ipsilaterally as part of the ventral corticospinal tract and decussates in the spinal cord prior to synapsing with the corresponding motoneurons (also called uncrossed ventral corticospinal tract). Some of the corticospinal neurons form instead the corticobulbar tract that sends input to brainstem motoneurons involved in the control of face, head, and neck muscles.

An injury at this level is generally associated with the pyramidal tract syndrome characterized by spasticity, paralysis, and loss of skilled movements.

On the other hand, cell bodies of the extrapyramidal tracts are mainly found in subcortical nuclei in the pons (reticular formation) and medulla oblongata. They send their axons in the spinal cord to motoneurons at all segmental levels. Specifically, the red, vestibular, tectum (superior colliculus), and reticular formation nuclei have cell bodies sending their axons through their respective descending extrapyramidal tract – the rubrospinal, vestibulospinal, tectospinal, and reticulospinal tracts. The former tract descends more laterally in the spinal cord whereas and the other three tracts travel ventrally (anterior column). The rubrospinal tract receives inputs from the motor and premotor areas 4 and 6. It sends projections to contralateral (decussation in the brain) motoneurons of the upper spinal cord segments for the control of both fine skilled movements and large powerful ones. It is generally considered to facilitate flexion and inhibit extension specifically in the upper extremities contralaterally. The tectospinal tract sends its axons in premotor lamina of the cervical spinal cord (VI–VIII) and is involved in neck and head motor control. In contrast with the other two tracks, the reticulospinal track sends projections to all levels of the spinal cord. It is involved in the control of autonomic functions (cardiovascular and respiratory functions, blood pressure, micturition, etc.). The vestibulospinal tract sends axons that travel either laterally or ventrally on the ipsilateral side of the spinal cord to premotor laminae VII and VIII. It is known to control muscular contraction levels specifically in extensors. The olivospinal tract originates in the inferior olivary nucleus and innervates contralaterally segments in the spinal cord.

Lesioned extrapyramidal tracts are associated with the extrapyramidal syndrome characterized by involuntary movements, muscular rigidity, and immobility without paralysis. Most of the descending tracts described above have roles to play normally (i.e., in absence of pathology or injury) in inducing and modulating stereotyped motor behaviors such as locomotion (Kandel et al., 2000; Guertin and Steuer, 2009; Guertin, 2013).

## Simple Reflex Pathways of the Gray Matter

As described above, white matter tracts serve essentially as “relays” for signals that carry descending (motor control) and ascending (sensory-related) information between the brain and spinal cord. In recent years, spinal cord physiologists have undertaken to characterize in greater details the cellular organization and function of neurons of the spinal gray matter. While it has been known for years now that some of those neurons form local spinal reflex pathways (so-called reflex arcs), others have been more recently identified as part of relatively complex circuits involved directly in the control of stereotyped motor behaviors – specifically locomotion. Other rhythmic motor behaviors associated with spinal cord networks (micturition, ejaculation, etc.) remain poorly characterized at least cellularly (Guertin and Steuer, 2009).

Simple reflex arcs or pathways are normally associated with classical reflexes. Activation of a simple reflex, in normal condition, typically leads to a rapid, predictable, repeatable, stereotyped, and involuntary motor reaction in response to the corresponding specific stimulus. Most reflexes are mediated locally, within one or two segments, by relatively simple neural pathways in the spinal cord – involving generally either one (monosynaptic), two (disynaptic), or more (polysynaptic) synapses and corresponding neurons (i.e., interneurons and motoneurons). Simple reflexes may be of autonomic (related with inner organs, eyes, blood vessels, etc.) or somatic (related with skeletal muscle responses) origin. The latter system has been more extensively studied mainly in cats. Among the known somatic spinal reflexes, the Ia, Ib, II, FRA (flexion reflex afferent) reflexes have been particularly well-characterized although their roles in the control of locomotion remain incompletely understood.

### Monosynaptic reflex

The so-called Ia monosynaptic reflex arc constitutes the best known somatic reflex pathway. It is also considered the simplest and fastest reflex of all. It mediates primary afferent (Ia) inputs from muscle spindles typically activated by muscle stretch (e.g., following a tendon jerk, tendon tap, or myotatic reflex). The Ia afferent input enters the spinal cord (dorsally via the corresponding spinal nerve) and establishes monosynaptic connections with homonymous alpha-motoneurons (e.g., soleus Ia afferent input sent to soleus motoneurons) in the gray matter (ventral horn area). This excitatory reflex is known to increase homonymous muscle contraction in response to muscle elongation. In addition to monosynaptic connections with the corresponding homonymous muscle, Ia afferents possess branches that also establish monosynaptic connections with synergistic alpha-motoneurons (e.g., soleus Ia afferent with gastrocnemius motoneurons). It is generally considered to play a role in tonus and postural adjustments. Although originally believed to be “non-modulatable,” evidence in recent years has shown that activity levels in the Ia monosynaptic arc can vary depending upon circumstances, phases, and tasks – e.g., amplitudes of the H-reflex (experimentally induced monosynaptic reflex mainly) decrease during walking compared with standing quietly. Also, during walking, H-reflex amplitudes are typically smaller during the swing phase (flexion) than the extension phase (extension; Stein and Capaday, 1988). An assessment of Ia reflex pathway function can provide clinically valuable information on neuropathological conditions. A reflex hammer stimulation eliciting an exaggerated (hyperreflexia) or diminished (hyporeflexia) monosynaptic reflex is generally considered as a sign of CNS or PNS damage, respectively (Matthews, 1972, 1991; Henneman, 1974). Although its role in CPG activity or, more generally in locomotion, remains unclear. However, findings made in a decerebrate and paralyzed cat model has provided evidence suggesting that Ia input elicited during locomotor activity [i.e., ENG-monitored fictive locomotor activity induced by electrical stimulation of the Mesencephalic Locomotor Region (MLR)] can contribute to further increase extensor activity during the stance phase. Given that it can also contribute to reset the rhythm during fictive locomotion, when activated during the swing phase, it was postulated that at least some Ia inputs uncovered only during locomotion may provide excitatory inputs to the CPG (specifically the extensor portion of the rhythm-generating core; Guertin et al., 1995). This short and rapid reflex action of Ia afferent input to the CPG may serve to compensate for an unsuspected increased of loading of the limb during ambulation as well as, more generally, to increase tonus in extensor muscles during stance (Gossard et al., 1994; Guertin et al., 1995; Pearson, 1995).

### Ib reflex pathways

Also known as the inverse myotatic reflex or autogenic inhibitory reflex pathway, the Ib reflex arc is associated with peripheral afferent inputs from the corresponding group Ib afferent fibers (Golgi tendon organs). It has been described initially as inhibiting the homonymous and synergistic alpha-motoneurons at rest although this specific inhibitory action may remain quiescent (i.e., its specific autogenic inhibitory action) in other conditions such as during locomotion. Inputs from the Ib afferents enter the spinal cord and inhibit disynaptically (two synapses involving one inhibitory neuron called the Ib interneuron) alpha-motoneurons at rest. This inhibitory reflex was originally believed to act as a protective mechanism against excessive muscle contraction (Matthews, 1972). However, this view has been challenged since in normal individuals (e.g., not SCI), Golgi tendon organs and Ib reflex pathways have been found to mediate inputs throughout a wide range of muscle activity and load. As with the Ia reflex, the Ib autogenic inhibitory reflex has been found to undergo extensive task-dependent modulation. Actually, during locomotor activity, it was found to become silent and to be replaced by another Ib afferent-induced reflex pathway providing instead extensive excitation to homonymous and synergistic alpha-motoneurons (at all joints of the lower extremities; Gossard et al., 1994; Guertin et al., 1995). The candidate excitatory interneurons mediating this disynaptic reflex is located in lumbar enlargement segments (premotor interneurons in lamina VII) and is rhythmically active during locomotion (Angel et al., 1996, 2005). Although the role of Ib autogenic inhibition remains unclear in resting conditions, its role during locomotion (i.e., the disynaptic excitatory reflex) may be, as with Ia inputs discussed earlier, to significantly enhance muscular contraction of extensors during the stance phase and to reset stepping to extension when activated during the swing phase (Guertin et al., 1995; Pearson, 1995). No clinically induced reflex movement has been associated with stimulation of Golgi tendon organs. However, the clasp knife reflex that was thought originally to be mediated only by autogenic inhibition is now believed to be the result of an interaction between Ia monosynaptic excitatory and Ib autogenic inhibitory actions on alpha-motoneurons in spastic patients (e.g., with injured descending tracts).

### Flexion (withdrawal) reflex pathways

Another well-described spinal reflex arc is the flexion reflex afferent (FRA) pathway activated by relatively high-threshold fibers (e.g., associated with cutaneous nociceptor A or C fibers, group II, III, and IV muscle afferent fibers, etc.). Ipsilaterally, it involves at least two interneurons (three or more synapses) in several segments of the spinal cord and alpha-motoneurons of several flexor muscles. It is also called the nocifensive reflex or withdrawal reflex pathway. Specifically, it comprises at least two excitatory interneurons (three synapses) for ipsilateral flexor motoneuron activation and two interneurons (one excitatory and one inhibitory) for ipsilateral extensor motoneuron inhibition. If the stimulus is particularly strong and intense, it may be accompanied by an activation of contralateral pathways and, thus, lead to the crossed-extension reflex producing concomitantly an extension of the contralateral limb or leg. Additional interneurons (and decussation) are involved in mediating the crossed-extension reflex – two excitatory interneurons for contralateral extensor motoneuron activation and two interneurons (one inhibitory and one excitatory) for contralateral flexor muscle inhibition. These reflex pathways are thought to play a role in withdrawal of a limb (unilateral flexion and contralateral extension) from a painful stimulus whereas the crossed-extension reflex would serve to enhance postural support during withdrawal of the affected limb from the painful stimulus. Task-dependent modulatory responses have also been found during locomotor activity since a long-lasting burst of activity is unraveled ipsilaterally during pharmacologically induced fictive locomotion (long-lasting FRA response). The corresponding interneurons have even been proposed to be part *per se* of the CPG for locomotion (specifically part of the flexor portion of a half-center organized-like network) since FRA stimulation under experimental conditions was shown to reset the step cycle to flexion (Jankowska et al., 1967a,b). Clinically, this reflex pathway, in resting conditions, may be investigated by induction of the Babinski sign (i.e., tongue depressor-induced plantar extension). Although normally found in infants, it is an indication of neurological problems in adults (e.g., spinal pyramidal tracts-induced injury caused by trauma or tumor). The long-lasting FRA response as well as myoclonus can also be uncovered following FRA stimulation in patients with SCI (Bussel et al., 1989; Swartz, 1998).

Although additional pathways have been associated with other “simple” reflex acts (e.g., reciprocal disynaptic inhibition, Renshaw recurrent inhibition, etc.), it is beyond the scope of this review to describe them all. Suffice to say that clinically, dysfunctional reciprocal inhibitory reflex pathways have been associated with increased co-contraction found in different pathological conditions such as Parkinson’s disease and spasticity.

## Complex Spinal Networks of the Gray Matter

### Early evidence and underlying concepts

To date, the best-characterized spinal network is undoubtedly the CPG for locomotion that directly controls the basic motor commands underlying ambulation. Originally believed to be of peripheral origin (former conclusion of Sir Charles Sherrington), it has been subsequently shown by his former student, Thomas Graham Brown, to be centrally located and composed of different sets of spinal interneurons (for a detailed description, see Guertin, 2009b). Thomas Graham Brown has indeed conducted experiments at the beginning of the twentieth century that, after a long hiatus, changed views on the neural control of locomotion (Graham Brown, 1914). His seminal work supported by subsequent evidence generated largely from the 1960s onward showed that, across species, rhythmic, and stereotyped motor behaviors including walking, flying, and swimming are controlled largely by a neuronal network generally referred to as CPG for locomotion.

Prior to that, earlier observations from paraplegic dogs revealed the existence of locomotor-like movements that can occur spontaneously some time after a complete transection (TX) of the spinal cord. That was elicited specifically when dropping one of the limbs from a flexed position (Flourens, 1824; Freusberg, 1874). Comparable observations by Philippson (1905) led him to conclude that the spinal cord controls locomotion using both central and reflex mechanisms. Sir Charles Sherrington’s (1910) work in TX cats and dogs provided additional evidence that such spinal locomotor-like movements were the result of reflex actions from proprioceptors onto some spinal centers. Indeed, in order to produce stepping movements in decerebrate, acutely spinal TX animals, they had to be lifted from the ground with the spine vertical and the hindlimbs pendent which, under their own weight, sufficed to elicit stepping that could be stopped by passively flexing one limb at the hip joint but not by cutting all corresponding cutaneous nerves. Sherrington already knew that such “involuntary” stepping movements were not solely the result of peripheral input mediated via the flexion and crossed-extension reflex pathways since passive immobilization of one hindlimb during vigorous stepping did not prevent stepping in the contralateral limb. However, it is Thomas Graham Brown, who described more directly the existence of a spinal neuronal network for the main neural commands underlying locomotion (see also Stuart and Hultborn, 2008 for a thorough description of Sherrington and Graham Brown’s original contributions). To summarize some of Graham Brown experiments, the animals, under general anesthesia, were lying on one side when stepping movements in the hindlimbs were spontaneously expressed (′narcosis progression′) after TX at the lower thoracic level. Since the level of anesthetic used was shown to abolish proprio- and extero-ceptive reflexes but not locomotor activity, Graham Brown proposed a “half-center” model constituted of two groups of spinal neurons reciprocally organized and mutually inhibiting each others that were capable of producing the basic rhythm and pattern for stepping. Activity in the first group of neurons (e.g., extensor half-center) would send motor commands to motoneurons (exciting extensors), and would inhibit simultaneously the reciprocal group of neurons (flexor half-center) preventing the excitation of antagonists (silencing flexors). After a period of “depression” (e.g., fatigue, adaptation, post-inhibitory rebound) of the extensor half-center, the flexor half-drive would predominate for a new phase of activity, etc.

However, it is only with the development of intracellular recordings that, in the 1960s, came the first evidence cellularly of its existence (half-center now generally referred to as the CPG; Jankowska et al., 1967a,b). A Swedish group led by Anders Lundberg recorded interneurons located in the lumbar segments of the cord (specifically in the lamina VII) that were active following FRA stimulation. Specifically, one group of neurons was found to be activated by ipsilateral FRA, a second group by contralateral FRA (coFRA), and a third group by both ipsi- and contralateral stimulation. After injection of l-DOPA and nialamide in TX cats, FRA stimulation evoked a high frequency burst followed by a long-lasting self-sustained series of discharges. One of the most important features was the reciprocal organization between these groups of interneurons since coFRA stimulation abolished the long-latency discharges evoked by ipsilateral FRA and vice versa. Finally, it was proposed that Ia interneurons (reciprocal Ia inhibition) could participate in the production of that locomotor pattern by receiving strong excitatory input from FRA interneurons given their corresponding rhythmic activity in l-DOPA-treated TX cats.

Since then, a variety of conceptual designs regarding its organization has been proposed. New models such as the hypothesis involving Renshaw cells, the ´ring´ model (Székely et al., 1969; Gurfinkel and Shik, 1973) to explain more complex locomotor patterns (e.g., backward vs. forward walking), the flexor burst generator (Duysens, 1977) with an asymmetrical excitatory drive from a flexor burst generator as well as the unit burst generator model (Grillner, 1981) with symmetrically organized burst generators (for each articulation or sets of muscles) even in absence of peripheral input. Other more recent models include the “synergy model” from Bizzi’s group (Tresch et al., 1999; Bizzi et al., 2008) and the bipartite model (or two-level CPGs) from McCrea’s group (Lafreniere-Roula and McCrea, 2005; McCrea and Rybak, 2008) inspired of ideas originally proposed by Perret and Cabelguen (1980).

Beyond these conceptual considerations on its organization, the CPG for locomotion has been identified as a group of interneurons localized, for most parts, in the lumbar area of the spinal cord. With an *in vitro* isolated spinal cord preparation from neonatal rats, Kjaerulff et al. (1994) used sulforhodamine-101, an activity-dependent marker/dye, to identify CPG neuron candidates in L1–L6 near the central canal as well as near the medial intermediate zone. A comparable approach used by Cina and Hochman (2000) showed the existence of a restricted number of labeled cells (presumably CPG neuron candidates) more specifically in L1–L5 segmental areas. These findings are supported also by other studies that showed, using electrical stimulation or selective lesions, key rhythmogenic CPG elements specifically in L1 and L2 in mice (Nishimaru et al., 2000). This is also supported by findings from Dimitrijevic et al. (1998) in SCI patients following epidural stimulation near L1–L2 that triggered locomotor-like movements in the lower extremities. Discrepancies may exist in other species regarding the exact localization of the CPG (e.g., in mid-lumbar segments in cats, Langlet et al., 2005). Some of the specific cellular elements possibly composing the CPG have been characterized genetically only recently (see subsequent sections, below).

## Recent Evidence of Other Complex Spinal Networks

Several other types of CPGs have also been found in the spinal cord – among them, CPGs involved in the basic control of scratching, micturition, and ejaculation (reviewed in Guertin and Steuer, 2009). Truitt and Coolen (2002) have provided clear evidence showing that ejaculation critically depends upon a network of neurons referred to as the Spinal Generator for Ejaculation or SGE. Located in the lumbar segments L3 and L4, the SGE was defined as a circuit capable of self-producing-sustained rhythmic output to motoneurons (pudendal motoneurons). The SGE was found to contain a key population of neurons (lumbar spino-thalamic neurons also called LSt cells) that (1) project to forebrain, (2) project to pudendal motoneurons (correspond to those located in the Onuf’s nucleus in men), and (3) receive input from sexual organs via the pudendal and dorsal nerve of the penis (Schroder, 1985; Truitt and Coolen, 2002). However, the type(s) of neurotransmitters involved and the subset of post-synaptic receptors associated with SGE activation and ejaculation remain largely unknown (recently reviewed in Courtois et al., 2012).

Micturition essentially depends, for the storage and periodic elimination of urine, on the coordinated activity of smooth and striated muscles in the several functional units of the lower urinary tract, namely the urinary bladder, the bladder neck, the urethra, and the urethral sphincter (Nadelhaft and Vera, 1995; Morrison et al., 2005). The coordination of these organs is also controlled by a complex network that is located partly in the spinal cord – the Spinal or Sacral Micturition Center (SMC). SMC neurons retrogradely labeled by injection of pseudorabies virus into the urinary bladder of the rat were found in regions receiving afferent input from the bladder (Nadelhaft and Vera, 1995; Sugaya et al., 2005). Localized both in the thoracolumbar (T8–9) and lumbosacral (L3–L4, L6–S1) areas of the spinal cord, electrostimulation of the corresponding areas in SCI subjects can produce episodes of voiding (reviewed in Courtois et al., 2012).

### Cellular elements that constitute the cpg for locomotion in animals

As mentioned earlier, the most extensively characterized complex network in the spinal cord remains, thus far, the CPG for locomotion. In primitive vertebrate species such as the lamprey, cellular components have been identified and even recorded from electrophysiologically nearly 30 years ago (LC cells, CC interneurons, etc.). Indeed, with its simpler neural system, it has been easier to investigate extensively neuronal activity from single spinal cells using the *in vitro* isolated spinal cord preparation from lampreys (reviewed in Grillner, 2006). The quest for dissecting further the CPGs has been supported also by findings obtained in parallel (or before) from non-mammalian and invertebrate species (see, e.g., Hugues and Wiersma, 1960; Delcomyn, 1977; Robertson and Pearson, 1985; Clarac and Pearlstein, 2007). In fact, studies on sea slugs, leeches, cockroaches, stick insects and crustacean locomotor (swimmeret), and motor (e.g., stomatogastric system) pattern-generating networks have played a pivotal role in understanding further the cellular and network bases of rhythmic motor and locomotor patterns in both invertebrate and vertebrate species (e.g., Hugues and Wiersma, 1960; Getting, 1977; Kristan and Weeks, 1983; Hopper and DiCaprio, 2004; Buschges et al., 2008).

Since the 1980s, pharmacological manipulations using a plethora of newly available receptor ligands (agonists and antagonists) have largely contributed to understand and further describe the detailed organization of the locomotor CPG. Experiments in *in vitro* isolated rodents (neonatal rat and mouse preparations) as well as, subsequently, in *in vivo* TX rodents (adult rat and mouse models) led to substantial advances in cellular target identification (receptors and channels) associated with locomotor rhythm-generation and modulation. Blood brain barrier permeable ligands have served to pharmacologically “dissect” *in vivo* the contribution of specific receptors and channels to locomotor rhythm-generation. Clear CPG-activating effects induced by specific drugs have also been found in various *in vitro* preparations from invertebrate and vertebrate species, although it is beyond the scope of this review to report on all of them (e.g., rats, Sigvardt et al., 1985; Cazalets et al., 1990; Cowley and Schmidt, 1994; mice, Nishimaru et al., 2000; Whelan et al., 2000; turtles, Guertin and Hounsgaard, 1998a).

As mentioned earlier, the noradrenergic system and specifically l-DOPA has been among the first systems to be associated with CPG activation in acutely TX cats and rabbits (e.g., Jankowska et al., 1967a,b; Viala and Buser, 1969; Grillner and Zangger, 1974; Pearson and Rossignol, 1991). Clonidine, an alpha-2 adrenergic receptor agonist, administered during sensory stimulation (e.g., tail or sexual organ pinching) was also reported to enhance the effects of training and/or sensory stimulation (remains unclear) on locomotor rhythmogenesis in TX cats (Forssberg and Grillner, 1973; Barbeau and Rossignol, 1991; Chau et al., 1998a,b). However, it has been difficult to determine site-specific actions (e.g., on motoneurons, CPG neurons, or primary afferents) from results in many of these earlier studies that were not designed to specifically assess drug-induced CPG activation *per se* (i.e., given the use of additional stimuli including tail stimulation, sexual organ pinching, regular training, or weight-support assistance).

More recently, experiments conducted in our laboratory using a simple and reliable semi-quantitative assay (ACOS, Guertin, 2005) and a mouse model (a complete low-thoracic TX) with no assistance or additional stimuli (e.g., no training, no tail stimulation, no sexual organ pinching, and no weight-support assistance to avoid unspecific non-drug-induced effects) have contributed to identify clearly a subset of transmembranal receptors involved in pharmacologically elicited, CPG-mediated locomotor-like movements in the lower extremities. For instance, l-DOPA, serotonin (5-HT) or dopamine (DA) receptor ligands such as 8-OH-DPAT, buspirone (5-HT1A/7 agonists), quipazine (5-HT2A/2C agonist), and SKF-81297 (D1-like agonist) were found to trigger significant locomotor-like movements (i.e., rhythmic bilaterally alternating flexions and extensions involving one or several hindlimb joints) whereas others ligands such as TFMPP (5-HT1B), m-CPP (5-HT2B/2C), SR57227A (5-HT3), or clonidine (adrenergic alpha-2 agonist) were shown to elicit mainly non-locomotor movements (i.e., non-bilaterally alternating movements, twitches, cramps, etc.) in TX mice (Guertin, 2004; Landry and Guertin, 2004; Landry et al., 2006a; Lapointe et al., 2008, 2009).

Using selective antagonists and genetically manipulated animals (e.g., 5-HT7KO mice), it has been clearly established that NMDA, 5-HT1, 5-HT7, 5-HT2A, and D1 receptors were specifically involved in mediating such CPG-activating locomotor-like effects (e.g., Landry et al., 2006a; Ung et al., 2008). For instance, endogenous glutamate release and NMDA receptor activation were reported as critically important for quipazine-induced effects since a complete loss of induced movement was found in NMDA antagonist (MK-801)-treated animals previously pretreated with NMDA (Guertin, 2004). Regarding DA receptors, administration of D2, D3, or D4 agonists was found not to generate significant hindlimb locomotor-like movements whereas D1/D5 agonists such as SKF-81297 can potently elicit locomotor-like movements that are lost in selective D1-like (D1/D5) antagonist-pretreated TX mice but no in D5^−/−^ paraplegic TX mice suggesting a specific contribution of the D1 subtype to CPG activation (Lapointe et al., 2009). All and all, pharmacological approaches in *in vivo*, untrained, and non-assisted TX animals have contributed to identify a subset of CPG-activating compounds (and corresponding receptors confirmed with selective antagonists and knockout animal models).

However, none of these molecules were found to generate large amplitude weight-bearing stepping movements *per se* in untrained, non-assisted, and non-sensory stimulated TX animals suggesting that only partial CPG-activating effects can be achieved using these ligands administered separately (Guertin, 2009a).

Subsequent studies conducted in our laboratory have shown that only simultaneous activation of some of these candidate receptors can, using similar pharmacological approaches and animal models, induce full locomotor-inducing effects. Partial CPG-activating effects (i.e., associated with crawling rather than full weight-bearing stepping) induced by some ligands, as mentioned above, were found indeed to turn into full CPG-activating effects (i.e., weight-bearing stepping in non-assisted, untrained, and non-stimulated paraplegic animals) by simultaneously administrating 8-OH-DPAT, quipazine, l-DOPA, and SKF-81293 (Guertin, 2008, 2009a). This said, the extent to which the cellular network activated pharmacologically *in vivo* corresponds to already identified CPG neuron candidates (electrophysiologically or genetically) remains unclear (see section below). However, dual immunohistochemical experiments recently showed locomotor activity-labeled (c-fos) 5-HT1A-, 5-HT2A-, or 5-HT7- positive neurons in the cat lumbar cord (specifically in laminae VII–VIII, Noga et al., unpublished data) suggesting that some of the locomotor activity-related receptors identified recently in *in vivo* models may indeed be located on CPG neurons.

Prior to these findings, numerous pharmacological studies aimed at improving locomotor function recovery after SCI have been also conducted in other *in vivo* models of SCI (e.g., in TX cats). However, it has remained difficult to consider those results as evidence of cellular targets associated with CPG activation or modulation given the used paradigm (experiments conducted during tail stimulation, sex organ pinching, body-weight supported, movement-assisted manually, etc.; e.g., Rossignol et al., 2001).

In recent years, advances in genetics and transgenic murine models have largely contributed to the identification of specific CPG neuron candidates *per se*. Several populations of interneurons involved in locomotor activity were indeed characterized genetically (e.g., V0–V3). One of them is the population of V0 interneurons that was associated with left-right alternation since mice without V0 interneurons (lacking the transcriptional factor Dbx1) displayed bilateral synchrony rather than bilateral alternation during locomotion (Lanuza et al., 2004). Another population referred to as the V1 inhibitory interneurons (expressing the transcription factor Engrailed 1) was associated with high locomotor frequencies since slow rhythms were found in En1-DTA mice (Gosgnach et al., 2006). Other genetically identified populations include the Chx10-expressing cells (V2a glutamatergic and V2b gabaergic interneurons, Lundfald et al., 2007) located in the intermediate zone of the gray matter in the lumbar spinal cord. These neurons were associated with frequency, amplitude, and bilateral coordination since all of these parameters were affected in Chx10-DTA mice (lacking V2a interneurons, Crone et al., 2008). V3 interneurons (Sim-1 expressing cells) constitute another recently identified population shown to participate in the production of a robust and balanced rhythm during locomotion. Indeed, rhythmic activity was found to be partially disrupted in mice lacking V3 interneurons (Zhang et al., 2008). Genetically engineered animals were also utilized to show that hopping instead of normal walking is displayed in mice lacking the ephA4 receptor-expressing lumbar interneurons (Butt et al., 2002; Kullander et al., 2003). Finally, another population of CPG neuron candidate called HB9 neurons was reported to provide neuronal excitation during locomotion. Although, no corresponding knockout model has been tested, compelling evidence suggests that the HB9 excitatory interneuron belongs to an asymmetrically organized rhythm-generating network (Wilson et al., 2005; Brownstone and Wilson, 2008).

Taken altogether, results from these exciting new studies in mice suggest that these genetically characterized interneurons (V0–V3, EphA4, HB9) may constitute different cellular components of the CPG. For instance, V0 interneurons form many cell types including commissural neurons which could establish inhibitory reciprocal connections between the two sides of the spinal cord. In turn, V1 interneurons may be associated with inhibitory interneurons such as the Ia inhibitory and Renshaw cells whereas HB9s may be excitatory interneurons constituting at least part of the rhythm-generating layer. However, additional studies need to be conducted in order to fully characterize these promising new CPG neuron candidates (Brownstone and Wilson, 2008; Goulding, 2009). It is important to mention also that using KO models none of these deletions has ever led to the elimination of rhythmic motoneuron excitation or rhythmic inhibition suggesting that several CPG secrets remain to be found and described. Note also that, using different approaches (i.e., electrophysiology) other CPG neuron candidates (e.g., Ib interneurons, FRA, commissural interneurons, Ia reciprocal interneurons, etc.) have also been characterized in cats and rodents (for reviews, see Jankowska, 2008; Guertin, 2009a).

### Particular cellular properties of the locomotor network

This is also an aspect of the CPG that remains incompletely understood. It is generally accepted that one of the main cellular features expected to be found in CPG neuron candidates is its capacity to express endogenously rhythmic activity during fictive or real locomotion. For several decades, it was also generally accepted that some neurons of the CPG were expected to express specifically autorhythmic or pacemaker-like properties given that locomotion is, by definition, a rhythmic motor behavior.

There have been indeed several cell populations in the corresponding area of the CPG (i.e., lumbar segments in mammalian species) shown in the last few decades to be capable of expressing pacemaker-like properties in specific conditions such as in the presence of NMDA alone or combined, at lower dose, with serotonin and DA (e.g., bath-applied in the case of *in vitro* isolated spinal cord preparations). Plateau potential is another intrinsic property believed by some researchers to be associated with endogenous (tetrodotoxin-resistant) pacemaker-like activity (e.g., Hochman et al., 1994; MacLean et al., 1997; Wang et al., 2006). Transmembranal currents and channels underlying both of these properties (voltage oscillations, NMDA ionophore, l-type (CaV1.3) Ca^2+^ channel, persistent Na^2+^ current, *I*_h_ current, *I*_A_ current, *I*_CAN_, *I*_NaP_ current, *I*_K(Ca)_ current, post-inhibitory rebound, reviewed in Harris-Warrick, 2002) have been relatively well-characterized in lamprey, turtle, tadpole, rodent, and cat preparations with some species-dependent specificities (Grillner and Wallén, 1985; Guertin and Hounsgaard, 1998b; Reith and Sillar, 1998; Brocard et al., 2010; extensively reviewed in:). Nonetheless, the specific contribution of these properties (in motoneurons and some interneurons) to real life locomotion remains speculative and a source of debate.

For further details, refer yourself to specialized review articles on that matter (Eken et al., 1989; Schmidt et al., 1998; Schmidt and Jordan, 2000; Grob and Guertin, 2007; Brocard et al., 2010).

## CPG for Locomotion in Humans

Elements composing the gray matter and some of its neural circuits have been preliminarily unraveled in animal studies, as described above. Thus far, there is compelling evidence that comparable systems exist in all vertebrate species including in humans. In fact, comparisons between locomotor activities, presumably CPG-driven, appear to be comparable in all studied species from invertebrates to vertebrates (Pearson, 1993). Despite the fact that locomotion is expressed differently in birds, fish, quadrupeds, or humans, evidence suggests that shared neuronal systems exist and have been well-preserved throughout evolution. This said, human plantigrade gait has been shown to display unique features – e.g., heel-strike at initial contact, a loading response in early stance, and asynchronized activity of lower-extremity extensor and flexor muscles (e.g., Forssberg and Hirschfeld, 1988; Stokes et al., 1989). Consequently, it has been postulated that human gait may, to some extent, be associated with a partly different organization of the CPG – e.g., with more extensive supraspinal influences over the regulation of locomotion compared with lower vertebrates.

Despite convincing evidence of a CPG in primates (e.g., Fedirchuk et al., 1998), only limited possibilities exist of directly demonstrating its existence in man. Indeed, concluding and undisputable evidence of its existence (as in TX and deafferented animal models – e.g., Grillner, 1981) in human beings is bound to fail given the inability, or unlikelihood, to ever being able of conducting experiments in volunteers displaying a perfectly isolated CPG *in vivo* – i.e., with no descending and peripheral inputs to lumbosacral segments.

On the other hand, to my knowledge, there has been no evidence of its lack of existence in humans. In fact, all evidence, although often indirect, points toward its phylogenetic preservation in humans as suggested by findings in patients with chronic SCI. Calancie et al. (1994) showed that involuntary lower-extremity stepping-like movements were expressed spontaneously in a subject with an incomplete cervical SCI. The movements expressed in a supine position were rhythmic, alternating, and forceful, involving all muscles of the legs bilaterally. However given the incompleteness of the lesion, this was not considered by most neurophysiologists as a concluding evidence of a CPG in humans. Calancie (2006) has also reported subsequently rhythmic movements (myoclonus) in a patient with complete SCI. An interesting case was reported by Nadeau et al. (2010), this time in a completely SCI patient who expressed EMG-recorded movements occurring spontaneously and characterized occasionally by coordinated (e.g., between flexors and extensors) rhythmic patterns of activity resembling bipedal stepping.

The best evidence has undoubtedly been provided by Dimitrijevic et al. (1998) who conducted epidural electrical stimulation of the spinal cord, below injury level. Such stimulation at the upper lumbar cord level (near L1–L2) was found to trigger stepping-like movements in completely SCI patients lying in a bed. These observations, combined with those from earlier reports (often anecdotal ones) of spontaneous rhythmic movements in the legs of patients with chronic SCI (Holmes, 1915; Lhermitte, 1919; Kuhn, 1950; Bussel et al., 1988, 1992), provide altogether compelling evidence for the existence of a CPG for locomotion in humans.

In contrast, some recovery of voluntary ambulation reported in incompletely SCI patients undergoing Body-Weight Supported Treadmill Training (BWSTT) can not be considered as valuable evidence for the existence of a CPG in humans given that improvement may be attributed largely to modulation of simple local reflex arcs activated cyclically during training *per se* (and modulated overtime by training) and/or pharmacological substances (e.g., baclofen, cyproheptadine, clonidine, etc.; Fung et al., 1990; Rémy-Néris et al., 1999; Knikou et al., 2009). In itself, each step being largely produced by manual assistance from physiotherapists is likely to trigger cyclically phasic group I excitation as well as other afferent reflexes (joint angle at the hip, group II from flexor, long-lasting FRAs) which can trigger, modulate, and reset CPG activity as shown in animal models (see section on simple reflex pathways).

## Role of Afferent and Descending Inputs on Locomotion

This section could in itself constitute a separate review article given the substantial body of data existing on that matter. Only a brief overview will be presented here.

First of all, the role of primary afferent inputs during locomotor activity is incompletely understood. However, it makes no doubt, in non-pathological cases, that the control system requires those inputs for successful progression in real life conditions. This said, results obtained from TX and deafferented animal models have clearly shown that the spinal cord is capable of generating rhythmic locomotor-like output and corresponding movements in the absence of descending and peripheral signals (Grillner, 1981). Graham Brown said – “There can be no question of its (proprioceptors) importance nor its suitability to augment the central mechanisms…. Its part must be regulative not causative” (Graham Brown, 1911). This notion still holds – sensory feedback is apparently important in modulating and adapting CPG-generated motor output for its adaptation to environmental constraints or obstacles. As summarized earlier in this review, inputs from Ia and Ib afferent fibers (so-called group I) during locomotion (or fictive locomotion) were shown, in reduced animal models, to increase up to 50% extensor activity as well as to reset CPG-mediated step cycle to a new extension (Guertin et al., 1995; Pearson, 1995). Group II inputs from flexors (Perreault et al., 1995) may also promote extension whereas long-lasting FRAs (although discrepancies may exist among all subtypes of FRAs) uncovered during locomotor activity can reset the step cycle to a new flexion (Jankowska et al., 1967a,b; Schomburg et al., 1998). In some experimental conditions, specific afferent inputs (e.g., sacral nerve stimulation, tail stimulation) can even trigger bouts of CPG-mediated activity (Lev-Tov et al., 2010; Zhang et al., 2010; Hochman et al., 2012; Liu et al., 2012) or significantly improve the long-lasting effects of regular treadmill training on involuntary stepping generation (Lovely et al., 1986; Bélanger et al., 1996).

In chronic SCI patients, the development of exaggerated reflexes (hyperreflexia and spasticity) may sometimes contribute to impair the remaining walking capabilities whereas, in other case, spasticity is believed helpful to promote or trigger the swing or flexion phase (Visintin and Barbeau, 1989; Wainberg et al., 1990; reviewed recently in Dietz and Sinkjaer, 2012).

As with afferent inputs, it remains unclear what the exact roles and interactions are between all brain structures and CPG neurons. This said, it is generally accepted that several areas of the brain are capable of inducing and/or modulating CPG-driven activity. For instance, Orlovsky et al. (1966) conducted experiments that demonstrated in decerebrate cats, receiving repetitive electrical stimulation of the brain stem, that locomotor-like activity can be induced and that the speed and mode of locomotion (i.e., walking, trotting, galloping) can depend upon the strength of brain (e.g., MLR) stimulation (Orlovsky et al., 1966; Orlovsky and Shik, 1976). In contrast, other studies have also shown that locomotion in cats, that have had the cerebral cortex removed as neonates, was purposeful and similar in pattern to that of intact animals (Villablanca et al., 1993). Other areas and descending tracts have been shown to be involved in locomotor rhythm-generation in spinally intact animals [thoroughly reviewed in Jordan et al., 2008; e.g., the medial longitudinal fasciculus (MLF), parapyramidal region (PPR), and cells originating in the medial pontomedullary reticular formation, A11 cells from the hypothalamus, the lateral vestibulospinal tract, and the lateral vestibular (Deiters’ nucleus)]. Given its well-characterized role in equilibrium, the cerebellum is also believed to be pivotal for bipedal locomotion. Ablation studies or genetic defect [e.g., Uner Tan Syndrome (UTS)] provide a clear demonstration of the cerebellum’s role in preserving the integrity of locomotor pattern generation (Roberts et al., 1992; Matsumoto et al., 2007) as well as in modulating extensor or flexor activity levels but not timing during locomotion (English, 1985; Molinari and Petrosini, 1993). However, modulation of locomotor activity level and timing may, in turn, be affected by activation of other areas such as the corticospinal tracts (e.g., pyramidal, Bretzner and Drew, 2005).

Several other studies have been conducted to investigate further the role of afferent and descending inputs during locomotion. Although, it is be beyond the scope of this article to review them further, insightful reviews have been published recently on their potential roles and related-central adaptations (Jordan et al., 2008; Rossignol et al., 2008; Barthélemy et al., 2011; Le Ray et al., 2011).

## Plasticity of CPG Neurons

There is clear evidence behaviorally suggesting that the spinal locomotor network may undergo adaptations and plasticity changes following a cervical or thoracic SCI. As described earlier, the spontaneous expression, within weeks or months post-SCI, of rhythmic movements (myoclonus or locomotor-like movements) in animals and humans constitute a clear sign of such phenomenon (Calancie et al., 1994; Guertin, 2005; Calancie, 2006; Ung et al., 2007; Nadeau et al., 2010).

However, at the cellular level, only indirect evidence of such adaptation has been found. In TX animal models, some immediate early genes (IEGs) were found to display up- or down-regulated expression levels in sublesional spinal cord areas within a few hours to a few days post-trauma. Landry et al. (2006b) reported in low-thoracic TX mice that *c-fos* and *nor-1* were respectively increased and decreased in L1–L2 dorsal horn and intermediate zone areas. Increased fos-immunoreactive levels were also found as soon as at 2.5 h post-trauma in sublesional segments (low-thoracic and lumbar laminae I–IV, VII, VIII, and X) of high-thoracic or cervical TX rats (Ruggiero et al., 1997). Because these same lumbar cord segments (specifically L1–L2 in mice) have been shown to contain critical CPG elements (Nishimaru et al., 2000) and, given that IEGs are well-known for their role in CNS development and plasticity (Ginty et al., 1992), spontaneous changes in IEG expression (i.e., specifically *c-fos* and *nor-1*) in L1–L2 segments may possibly be considered as among the first cellular events associated with cell property changes and increased CPG excitability post-SCI (see discussion in Landry et al., 2006b).

Numerous regulatory changes are likely to be subsequently activated after IEG alterations. These may include tumor necrosis factor-alpha (TNF-α) and preprodynorphin because their expression was found to display increased levels in lumbar segments several hours to several days post-TX (Yakovlev and Faden, 1994). Increased levels of nitric oxide synthase (NOS) expression were also found in lumbar segments at 1 and 7 days in low-thoracic hemisected rats (Lukacova et al., 2006). Byrnes et al. (2006) also reported changes in gene expression [C1qb, Galectin-3, and p22(phox)] distally from the epicenter that peak at 3 and 7 days in mild, moderate, or severe SCI rats. Substantial changes in gene expression pattern have been reported several days to several weeks after injury in the whole spinal cord (Ma et al., 2006) or in areas above the lesion (Schmitt et al., 2006). Although a precise role for most of these regulatory and gene expression changes in functional recovery and CPG activity or dysfunction remains unknown, scientists generally agree that an association may be made between such alterations, plasticity, reorganization, and cell property changes in distally located networks such as the CPG (Chi et al., 1993; Yakovlev and Faden, 1994; Ruggiero et al., 1997). A role for some of these changes (e.g., with TNF-α) in neuropathic pain (e.g., Peng et al., 2006) or inflammation (e.g., Pineau and Lacroix, 2007) also exist. Other good candidates for plasticity and increased CPG excitability include glycine receptors (increased expression) and GAD-67 (enzyme in γ-aminobutyric acid synthesis) which expression levels are changed in CPG-related areas post-TX (Edgerton et al., 2001; Tillakaratne et al., 2002). Increased 5-HT_1A/7_ receptor expression in lumbar spinal cord segments of chronic paraplegic cats (Giroux et al., 1999) and increased α_1_ and α_2_ adrenoceptors autoradiographic levels were also observed at sublesional levels in the spinal cord (Giroux et al., 1999). Finally, growth factors and neurotrophic factors such as NGF, BDNF, and NT-3 were found to display up-regulated levels of expression in sublesional areas of T9/10 transected rats (Li et al., 2007) although discrepancies exist (Gomez-Pinilla et al., 2004).

## Abnormal or Pathological Rhythmic Leg Movements

Clinically, there has been several pathologies and neurological disorders associated presumably with abnormal or deregulated CPG activities. Considering all the different CPGs located throughout the CNS that are involved in mediating various biological rhythms (e.g., sleep, mastication, ocular movement, breathing, deglutition, etc.), several signs, or pathological complications associated with rhythm-generator problems have been reported over the years. For instance, nystagmus or involuntary rhythmic eye movement is an ocular problem that is associated with heredity problems, poor vision, cortical blindness, intracranial neoplasms, or cerebellar ataxia. Pending upon the specific cause, different types of treatments and corresponding rhythmicity or “pacemaker” problems have been proposed (Strupp et al., 2011). Patients suffering of Amyotrophic Lateral Sclerosis (ALS) often encounter deglutition and swallowing problems. The latter, normally controlled by the swallowing CPG located in the bulbar area of the brain, has been shown to undergo dysfunctional activity when corticobulbar control becomes progressively impaired in ALS patients (Aydogdu et al., 2011). For patients with Rett Syndrome, a dysfunction of the CPG for breathing associated with a mutation in the methyl-CpG binding protein2 (MECP2 gene) ends up affecting rhythm-generating networks and breathing complications. Although no drug treatment exists for such swallowing rhythmic problems, NE reuptake inhibitors (e.g., desipramine) have been shown to reduce respiratory rhythmic problems in animal models of Rett Syndrome (Roux et al., 2007). Several biological rhythmic problems may occur in patients suffering of an injury to the pons. The latter is an area that has been proposed to play a pivotal in mediating several biological rhythms – e.g., a patient suffering of a hemorrhagic lesion in that area was reported to display synchronous rather than rhythmic motor activities of the eye, tongue, mandible, pharynx, diaphragms, and other muscles in which myoclonus. Myoclonus, involuntary rhythmic leg movements, and other spontaneously occurring and stereotyped rhythmic movements have also been reported in patients with Multiple Sclerosis (MS) or SCI (Yokota et al., 1991; Shneyder et al., 2011). A systematic review has shown recently that Restless Leg Syndrome (RLS) prevalence amongst patients with MS ranges from 12.12 to 57.50% in different populations (Schürks and Bussfeld, 2012). There is some evidence suggesting that RLS, Alternating Leg Muscle Activation (ALMA) or Periodic Limb Movements during Sleep (PLMS), found in individuals with SCI, MS, sleep disorders, and other types of neurological problems, may be associated with abnormal and involuntary CPG activation (Chervin et al., 2003; Tassinari et al., 2005, 2009; Consentino et al., 2006). This said, many of these pathological complications are generally considered to be associated with several mechanisms beyond a malfunction of the CPG (e.g., for RLS, role for DA imbalance, iron deficiency, etc., Thorpe et al., 2011; Jones and Cavanna, 2012; Rye and Trotti, 2012).

In contrast with most of these neurological problems where spontaneous and abnormal CPG activity have been proposed as one of the underlying mechanisms, a recently identified, rare and fascinating neurological condition described by Dr. Uner Tan has been instead associated with a entirely different and unique aspect of CPG malfunction. It is called UTS and is essentially characterized by the expression of quadrupedal gait in humans. It is typically accompanied with flexed head and body, primitive speech, severe mental retardation, and cerebellar problems. UTS has been originally described as an autosomal-recessively inherited disease, found initially in 5 of 19 children from the same Turkish family in 2006 (Tan, 2006a). Although it was proposed to correspond to a genetic mutation affecting the normal development of bipedalism during childhood, the underlying mechanisms remain unclear. Since then, several other families have been found to carry similar locomotion impairments. In all cases, those individuals walk on their wrists and feet with straight legs and arms (Tan, 2006b) and display ataxia and cerebellar atrophy (e.g., vermial hypoplasia as shown by MRI and PET scans, Tan, 2008; Tan et al., 2008) that is associated with a missense mutation in the ATPase, aminophospholipid transporter protein ATP8A2 mainly expressed in the cerebellum (Emre Onat et al., 2012). Interestingly, other genetic diseases involving CPG deregulations such as in Rett Syndrome have also been shown to be associated with multiple complications including mental retardation (Johnston et al., 2003). Observations from Dr Tan have led him to propose that spinal locomotor neurons (e.g., CPG) may contribute to this disorder as these individuals exhibit ancestral traits of locomotion – that is quadrupedal gait with rudimentary intelligence and primitive speech which, in turn, may be associated with devolution or reverse evolution. However, as interesting has it may sound, it is rather speculative and detailed mechanisms presumably underlying such abnormal (or primitive) CPG activities remain unclear and based largely upon behavioral observations and indirect associations with locomotion features in other species.

Dr Tan’s theory is nonetheless supported by results from independent laboratories providing solid evidence for the existence in humans of another CPG involved instead in the control of rhythmic arm movements – reminiscent of what is found in quadrupeds. Indeed, neuronal coupling between upper (cervical) and lower (lumbar) locomotor CPGs has been shown in humans to undergo a decrease in coupling strength during childhood and the development of bipedality. The shared or coupled CPG hypothesis has been first postulated by Grillner (1981) to explain swimming movement in lampreys. The locomotor network in lower vertebrates consists indeed of multiple spinal CPGs, with descending pathways activating individual CPGs for selective control of various segments in lampreys or joints and muscle groups in the case of terrestrial species. Coordinated movement within a limb could be achieved through phase-dependent interactions of different CPGs controlling that limb according to Grillner’s model (e.g., between hip and knee CPGs). Evidence of such an existing organization in humans has been provided by Zehr and colleagues who evaluated the effect of small leg displacements during gait on leg and arm EMG activity during walking. During progression on a split-belt treadmill (velocity 3.5 km/h), short accelerations or decelerations were randomly applied to the right belt during the mid or end stance phase whereas trains of electrical stimuli were delivered to the right distal tibial nerve. The results showed task-dependent and flexible neuronal coupling activities between lower and upper limb muscles in humans suggesting that several CPGs (e.g., for arms vs for leg rhythmic movements) for walking are still functional in the human lumbar and cervical spinal cord areas. These results are also compatible with the assumption that proximal arm muscle responses are associated with the swinging of the arms during gait, as a residual function of quadrupedal locomotion, as proposed by Dietz and colleagues. This organization and coupling relationship are also supported by data from *in vitro* isolated spinal cord preparations (Juvin et al., 2012).

All in all, given these evidence and findings, it is not unreasonable to postulate that impaired equilibrium that may be directly attributed to cerebellar atrophy and deficits in UTS patients could have enabled the expression of more tightly coupled activities between the upper and lower CPGs for the development of quadrupedal walking instead of bipedal locomotion.

One of the questions then is why cerebellar deficits lead to stronger CPG coupling and quadrupedal walking? Is it simply that insufficient equilibrium can not allow bipedal walking to be expressed? Given that the vermis is particularly affected by the missense mutation, it is probable that impaired activity along the vestibulospinal tract is also found. As described earlier, this descending tract is well-known to regulate leg extensor activity, which is obviously considerably important for stability and equilibrium during bipedal locomotion. Therefore, in absence of sufficient drive to extensor muscles of the legs (prerequisite to weight-bearing stepping), corresponding sublesional plasticity changes may have occurred as a compensatory mechanism for preserving, at least, the expression of some form of CPG activation and walking capabilities. Plasticity changes that may be found are probably similar, to some extent, with those described in animal models of SCI (sublesional increase in CPG areas of c-fos, specific 5-HT1A receptors, NA receptors, and neurotrophic factor expression levels). Ancestral upper CPG activity, enabled or uncovered by this pathological condition, would not restore equilibrium but at least some descending drive (propriospinal instead of supraspinal) capable to support some leg movements during progression with all four extremities. Based upon these hypotheses, the next question is: Can specific therapies be proposed for restoring normal CPG coupling and activity and corresponding bipedal walking in UTS patients? Although unlikely to be proposed in a near future as a standard therapy, it is obvious that cell replacement and grafting approaches that can repair the cerebellum of UTS patients would constitute an ideal solution for the recovery of bipedal walking. In the meantime, other approaches, strategies, and cellular targets could possibly be proposed and explored. One of them is obviously associated with means that can improve vestibulospinal control over extensor activity in the lower limbs. Electrical stimulation of extrapyramidal tracts (e.g., vestibulospinal tracts) or corresponding nuclei for improving or restoring posture and equilibrium capabilities, as shown in cats (e.g., Orlovsky et al., 1966; Orlovsky and Shik, 1976), can probably constitute an interesting therapeutic avenue for UTS patients. Another avenue to increase lower CPG drive and extensor activity may be based on electrical stimulation of ventrolateral tracts near thoracic segmental areas already known for reactivating the lumbar CPG in animal models (Cheng and Magnuson, 2011). Alternatively, direct stimulation of the CPG (e.g., intraspinally, epidurally, or transcutaneously, Holinski et al., 2011; Gorodnichev et al., 2012; Moshonkina et al., 2012) or indirectly by peripheral nerve stimulation (e.g., Guertin et al., 1995; Ollivier-Lanvin et al., 2011) or muscle vibration (Field-Fote et al., 2012) can possibly be used to boost lumbar CPG activation and bipedal stepping expression. Pharmacological aids such as fast-acting and brain-permeable adrenergic α1 agonists (e.g., methoxamine or a regulatory-approved midodrine sold as Amatine) could also possibly help to increase leg extensor activity in UTS patients. Indeed, in TX cats, such drugs have been shown during treadmill training to significantly increase extensor activity in lower extremities without changing timing or coordination (Chau et al., 1998a). The transfer of skills and capabilities gained by electrical and/or pharmacological aids could advantageously be enhanced by specialized rehabilitation approaches such as those developed for incomplete SCI patients (e.g., BWSTT). Recent findings in animal models of SCI have shown that recovery of walking (presumably voluntary elicited) using drugs and BWSTT in bipedal mode can be enhanced even in quadrupeds (Sławińska et al., 2012; van den Brand et al., 2012). In other words, quadrupeds may be made to display bipedal walking which strongly supports the idea that quadrupedal walking in UTS patients can also be transformed into bipedal walking under specific training conditions. As a support for lower CPG activation, other types of pharmacological aids could also be used. DAergic and serotonergic agonists and precursors, administered as a combination therapy, have been shown to synergistically activate the lumbar CPG for locomotion in TX animals (Lapointe and Guertin, 2008; Guertin et al., 2010). D1 receptors and 5-HT1A receptors have been specifically shown to mediate this synergistic effect (Landry et al., 2006a; Lapointe et al., 2009). Using such an approach, multiple deregulated systems (muscular, skeletal, vascular, hormonal, etc.) have even been shown to regain near normal function in chronic SCI animals (Guertin et al., 2011). A return to near normal condition has also been shown for at least some of the plasticity changes associated with CPG dysfunction (e.g., Tillakaratne et al., 2002). A particularly potent CPG-activating cocktail, called Spinalon™, is in fact currently in clinical development (Phase IIa) for safe and efficient reactivation of lumbar CPG neurons in SCI patients (www.NordicLifeSciencePipeline.com). If safety data are found, it will be of interest to eventually explore and develop such a treatment also for bipedal training of UTS patients. As discussed earlier in the review, several afferent pathways and simple reflex arcs have been shown to modulate stepping in real life conditions. This will perhaps require additional adjustments and specific pharmacological aids to separately control spasticity and other peripheral input problems that can impair normal bipedal ambulation in some pathological conditions (Fung et al., 1990).

## Concluding Remarks

Central pattern generators are complex structures for which many of the cellular elements have not yet been unraveled. Nonetheless, compelling evidence supports key roles in controlling biological rhythms such as locomotion in most if not all vertebrate species. Several neurological conditions and disorders displaying abnormal rhythmic and locomotor-like movements have been presumably associated with dysfunctional CPG activity. Although the causes are often complex and still incompletely understood in most cases, several research avenues have already been pursued (e.g., RLS, myoclonus) and could still be explored (UTS) to restore normal CPG activity and corresponding bipedal walking capabilities. The CPG for locomotion has clearly been shown to be both flexible and adaptable (e.g., Harris-Warrick, 2011) providing hope for the development in a near future of safe therapeutic approaches capable of re-establishing normal CPG activity.

## Conflict of Interest Statement

The author wishes to declare financial competing interests in Nordic Life Science Pipeline.

## Abbreviations

5-HT, serotonin; ALMA, alternating leg movement activation; CNS, central nervous system; CPG, central pattern generator; DA, dopamine; EMG, electromyogram; NMDA, *N*-methyl-d-aspartate; PLM, periodic leg movement; RLS, restless leg syndrome; SCI, spinal cord injury; TX, spinal cord transected; UTS, Uner Tan syndrome.

## References

[B1] Angel M. J. Guertin P. Jiménez I. McCrea D. A. (1996). Group I extensor afferents evoke disynaptic EPSPs in cat hindlimb extensor motorneurones during fictive locomotion. J. Physiol. 493, 851–861 8865080 10.1113/jphysiol.1996.sp021538 PMC1160683

[B2] Angel M. J. Jankowska E. McCrea D. A. (2005). Candidate interneurones mediating group I disynaptic EPSPs in extensor motoneurones during fictive locomotion in the cat. J. Physiol. (Lond.) 563(Pt 2), 597–610 10.1113/jphysiol.2004.076034 15618278 PMC1665583

[B3] Aydogdu I. Tanriverdi Z. Ertekin C. (2011). Dysfunction of bulbar central pattern generator in ALS patients with dysphagia during sequential deglutition. Clin. Neurophysiol. 122, 1219–1228 10.1016/j.clinph.2010.11.002 21111672

[B4] Barbeau H. Rossignol S. (1991). Initiation and modulation of the locomotor pattern in the adult chronic spinal cat by noradrenergic, serotonergic and dopaminergic drugs. Brain Res. 546, 250–260 10.1016/0006-8993(91)91489-N 2070262

[B5] Barthélemy D. Grey M. J. Nielsen J. B. Bouyer L. (2011). Involvement of the corticospinal tract in the control of human gait. Prog. Brain Res. 192, 181–197 10.1016/B978-0-444-53355-5.00012-9 21763526

[B6] Bélanger M. Drew T. Provencher J. Rossignol S. (1996). A comparison of treadmill locomotion in adult cats before and after spinal transection. J. Neurophysiol. 76, 471–491 8836238 10.1152/jn.1996.76.1.471

[B7] Bizzi E. Cheung V. C. d’Avella A. Saltiel P. Tresch M. (2008). Combining modules for movement. Brain Res. Rev. 57, 25–33 10.1016/j.brainresrev.2007.08.004 PMC4295773 18029291

[B8] Bretzner F. Drew T. (2005). Contribution of the motor cortex to the structure and the timing of hindlimb locomotion in the cat: a microstimulation study. J. Neurophysiol. 94, 657–672 10.1152/jn.01245.2004 15788518

[B9] Brocard F. Tazerart S. Vinay L. (2010). Do pacemakers drive the central pattern generator for locomotion in mammals? Neuroscientist 16, 139–155 10.1177/1073858409346339 20400712

[B10] Brownstone R. B. Wilson J. M. (2008). Strategies for delineating spinal locomotor-rhythm generating networks and the possible role of Hb9 interneurones in rhythmogenesis. Brain Res. Rev. 57, 64–76 10.1016/j.brainresrev.2007.06.025 17905441 PMC5061561

[B11] Buschges A. Akay T. Gabriel J. P. Schmidt J. (2008). Organizing network action for locomotion: insights from studying insect walking. Brain Res. Rev. 57, 162–171 10.1016/j.brainresrev.2007.06.028 17888515

[B12] Bussel B. Roby-Brami A. Azouvi P. (1992). “Organization of reflexes elicited by flexor reflex afferents in paraplegic man: evidence for a stepping generator,” in Muscle Afferents and Spinal Control of Movement, 1st Edn, eds Jami L. Pierrot-Deseilligny E. Zytnicki D. (Oxford: Pergamon), 427–432

[B13] Bussel B. Roby-Brami A. Yakovleff A. Bennis N. (1989). Late flexion reflex in paraplegic patients. Evidence for a spinal stepping generator. Brain Res. Bull. 22, 53–56 10.1016/0361-9230(89)90127-5 2653569

[B14] Bussel B. C. Roby-Brami A. Yakovleff A. Bennis N. (1988). “Evidences for the presence of a spinal stepping generator inpatients with a spinal cord section,” in Posture and Gait: Development, Adaptation and Modulation, eds Amblard B. Berthoz A. Clarac F. (Amsterdam: Elsevier), 273–278

[B15] Butt S. J. Harris-Warrick R. M. Kiehn O. (2002). Firing properties of identified interneuron populations in the mammalian hindlimb central pattern generator. J. Neurosci. 22, 9961–9971 12427853 10.1523/JNEUROSCI.22-22-09961.2002 PMC6757818

[B16] Byrnes K. R. Garay J. Di Giovanni S. De Biase A. Knoblach S. M. Hoffman E. P. (2006). Expression of two temporally distinct microglia-related gene clusters after spinal cord injury. Glia 53, 420–433 10.1002/glia.20295 16345062

[B17] Calancie B. (2006). Spinal myoclonus after spinal cord injury. J. Spinal Cord Med. 29, 413–424 17044393 10.1080/10790268.2006.11753891 PMC1864857

[B18] Calancie B. Needham-Shropshire B. Jacobs P. Willer K. Zych G. Green B. A. (1994). Involuntary stepping after chronic spinal cord injury. Evidence for a central rhythm generator for locomotion in man. Brain 117(Pt 5), 1143–1159 10.1093/brain/117.5.1143 7953595

[B19] Cazalets J. R. Grillner P. Menard I. Cremieux J. Clarac F. (1990). Two types of motor rhythm induced by NMDA and amines in an in vitro spinal cord preparation of neonatal rat. Neurosci. Lett. 111, 116–121 10.1016/0304-3940(90)90354-C 2186309

[B20] Chau C. Barbeau H. Rossignol S. (1998a). Effects of intrathecal alpha1- and alpha2-noradrenergic agonists and norepinephrine on locomotion in chronic spinal cats. J. Neurophysiol. 79, 2941–2963 9636099 10.1152/jn.1998.79.6.2941

[B21] Chau C. Barbeau H. Rossignol S. (1998b). Early locomotor training with clonidine in spinal cats. J. Neurophysiol. 79, 392–409 9425208 10.1152/jn.1998.79.1.392

[B22] Cheng J. Magnuson D. S. (2011). Initiation of segmental locomotor-like activities by stimulation of ventrolateral funiculus in the neonatal rat. Exp. Brain Res. 214, 151–161 10.1007/s00221-011-2816-7 21858680 PMC3192501

[B23] Chervin R. D. Consens F. B. Kutluay E. (2003). Alternating leg muscle activation during sleep and arousals: a new sleep-related motor phenomenon? Mov. Disord. 18, 551–559 10.1002/mds.10397 12722169

[B24] Chi S. I. Levine J. D. Basbaum A. I. (1993). Peripheral and central contributions to the persistent expression of spinal cord fos-like immunoreactivity produced by sciatic nerve transection in the rat. Brain Res. 617, 225–237 10.1016/0006-8993(93)91090-F 8402151

[B25] Cina C. Hochman S. (2000). Diffuse distribution of sulforhodamine-labeled neurons during serotonin-evoked locomotion in the neonatal rat thoracolumbar spinal cord. J. Comp. Neurol. 423, 590–602 10.1002/1096-9861(20000807)423:4<590::AID-CNE5>3.0.CO;2-L 10880990

[B26] Clarac F. Pearlstein E. (2007). Invertebrate preparations and their contribution to neurobiology in the second half of the 20th C. Brain Res. Rev. 54, 113–161 10.1016/j.brainresrev.2006.12.007 17500093

[B27] Consentino F. L. Iero I. Lanuzza B. Tripodi M. Ferri R. (2006). The neurophysiology of the alternating leg muscle activation (ALMA) during sleep: study of one patient before and after treatment with pramipexole. Sleep Med. 7, 63–71 10.1016/j.sleep.2006.07.153 16310410

[B28] Courtois F. Carrier S. Charvier K. Guertin P. A. Journel M. (2012). The control of male sexual responses. Curr. Pharmacol. Des. (in press) 10.2174/13816128113199990333 23360268

[B29] Cowley K. C. Schmidt B. J. (1994). A comparison of motor patterns induced by N-methyl-d-aspartate, acetylcholine and serotonin in the in vitro neonatal rat spinal cord. Neurosci. Lett. 171, 147–150 10.1016/0304-3940(94)90625-4 8084477

[B30] Crone S. A. Quinlan K. A. Zagoraiou L. Droho S. Restrepo C. E. Lundfald L. (2008). Genetic ablation of V2a ipsilateral interneurons disrupts left-right locomotor coordination in mammalian spinal cord. Neuron 60, 70–83 10.1016/j.neuron.2008.08.009 18940589

[B31] Delcomyn F. (1977). “Co-ordination of invertebrate locomotion,” in Mechanics and Energetics of Locomotion, eds Alexander R. M. Gold Spink G. (London: Chapman and Hall), 82–114

[B32] Dietz V. Sinkjaer T. (2012). Spasticity. Handb. Clin. Neurol. 109, 197–211 23098714 10.1016/B978-0-444-52137-8.00012-7

[B33] Dimitrijevic M. R. Gerasimenko Y. Pinter M. M. (1998). Evidence for a spinal central pattern generator in humans. Ann. N. Y. Acad. Sci. 860, 360–376 10.1111/j.1749-6632.1998.tb09062.x 9928325

[B34] Duysens J. (1977). Reflex control locomotion as revealed by stimulation of cutaneous afferents in spontaneously walking premammillary cats. J. Neurophysiol. 40, 737–751 886369 10.1152/jn.1977.40.4.737

[B35] Edgerton V. R. Leon R. D. Harkema S. J. Hodgson J. A. London N. Reinkensmeyer D. J. (2001). Retraining the injured spinal cord. J. Physiol. 533, 15–22 10.1111/j.1469-7793.2001.0015b.x 11351008 PMC2278598

[B36] Eken T. Hultborn H. Kiehn O. (1989). Possible functions of transmitter-controlled plateau potentials in alpha motoneurones. Prog. Brain Res. 80, 257–67; discussion 239–242. 10.1016/S0079-6123(08)62219-0 2699366

[B37] Emre Onat O. Gulsuner S. Bilguvar K. Nazli Basak A. Topaloglu H. Tan M. (2012). Missense mutation in the ATPase, aminophospholipid transporter protein ATP8A2 is associated with cerebellar atrophy and quadrupedal locomotion. Eur. J. Hum. Genet. 10.1038/ejhg.2012.170 22892528 PMC3573203

[B38] English A. W. (1985). Interlimb coordination during stepping in the cat: the role of the dorsal spinocerebellar tract. Exp. Neurol. 87, 96–108 10.1016/0014-4886(85)90136-0 3967704

[B39] Fedirchuk B. Nielsen J. Petersen N. Hultborn H. (1998). Pharmacologically evoked fictive motor patterns in the acutely spinalized marmoset monkey (Callithrix jacchus). Exp. Brain Res. 122, 351–361 10.1007/s002210050523 9808308

[B40] Field-Fote E. Ness L. L. Ionno M. (2012). Vibration elicits involuntary, step-like behavior in individual with spinal cord injury. Neurorehabil. Neural Repair 26, 861–869 10.1177/1545968311433603 22328683

[B41] Flourens M.-J.-P. (1824). *Recherches Expérimentales Sur Les Propriétés et les Fonctions du Système Nerveux, Dans les Animaux Vertébrés* [Experimental Studies on the Properties and Functions of the Nervous System in Vertebrate Animals]. Paris: Chez Crevot

[B42] Forssberg H. Grillner S. (1973). The locomotion of the acute spinal cat injected with clonidine i.v. Brain Res. 50, 184–186 10.1016/0006-8993(73)90606-9 4690545

[B43] Forssberg H. Hirschfeld H. (1988). Phasic modulation of postural activation patterns during human walking. Prog. Brain Res. 76, 221–227 10.1016/S0079-6123(08)64508-2 3064148

[B44] Freusberg A. (1874). Reflexbewegungen beim Hunde. Pflügers Arch. 9, 358–391 10.1007/BF01612347

[B45] Fung J. Stewart J. E. Barbeau H. (1990). The combined effects of clonidine and cyproheptadine with interactive training on the modulation of locomotion in spinal cord injured subjects. J. Neurol. Sci. 100, 85–93 10.1016/0022-510X(90)90017-H 2089144

[B46] Getting P. A. (1977). Neuronal organization of escape swimming in Tritonia. J. Comp. Physiol. 121, 325–342 10.1007/BF00613012

[B47] Ginty D. D. Bading H. Greenberg M. E. (1992). Trans-synaptic regulation of gene expression. Curr. Opin. Neurobiol. 2, 312–316 10.1016/0959-4388(92)90121-Z 1322751

[B48] Giroux N. Rossignol S. Reader T. A. (1999). Autoradiographic study of alpha1- and alpha2-noradrenergic and serotonin1A receptors in the spinal cord of normal and chronically transected cats. J. Comp. Neurol. 406, 402–414 10.1002/(SICI)1096-9861(19990412)406:3<402::AID-CNE8>3.3.CO;2-6 10102504

[B49] Gomez-Pinilla F. Ying Z. Roy R. R. Hodgson J. Edgerton V. R. (2004). Afferent input modulates neurotrophins and synaptic plasticity in the spinal cord. J. Neurophysiol. 92, 3423–3432 10.1152/jn.00432.2004 15548637

[B50] Gorodnichev R. M. Pivovarova E. A. Pukhov A. Moiseev S. A. Savokhin A. A. Moshonkina T. R. (2012). [Transcutaneous electrical stimulation of the spinal cord: non-invasive tool for activation of locomotor circuitry in human]. Fiziol. Cheloveka. 38, 46–56 22679796

[B51] Gosgnach S. Lanuza G. M. Butt S. J. Saueressig H. Zhang Y. Velasquez T. (2006). V1 spinal neurons regulate the speed of vertebrate locomotor outputs. Nature 440, 215–219 10.1038/nature04545 16525473

[B52] Gossard J. P. Brownstone R. M. Barajon I. Hultborn H. (1994). Transmission in a locomotor related group Ib pathway from hindlimb extensor muscles in the cat. Exp. Brain Res. 98, 213–228 10.1007/BF00228410 8050508

[B53] Goulding M. (2009). Circuits controlling vertebrate locomotion: moving in a new direction. Nat. Rev. Neurosci. 10, 507–518 10.1038/nrg2648 19543221 PMC2847453

[B54] Graham Brown T. (1911). The intrinsic factors in the act of progression in the mammal. Philos. Trans. R. Soc. Lond. B Biol. Sci. 84, 309–319

[B55] Graham Brown T. (1914). On the nature of the fundamental activity of the nervous centres; together with an analysis of the conditioning of rhythmic activity in progression, and a theory of the evolution of function in the nervous system. J. Physiol. 48, 18–46 16993247 10.1113/jphysiol.1914.sp001646 PMC1420503

[B56] Grillner S. (1981). “Control of locomotion in bipeds, tetrapods, and fish,” in Handbook of Physiology; The Nervous System II, eds Brookhart J. M. Mountcastle V. B. (Bethesda, MD: American Physiological Society), 1179–1236

[B57] Grillner S. (2006). Neuronal networks in motion from ion channels to behaviour. An. R. Acad. Nac. Med. (Madr.) 123, 297–298 17310636

[B58] Grillner S. Wallén P. (1985). The ionic mechanisms underlying N-methyl-d-aspartate receptor-induced, tetrodotoxin-resistant membrane potential oscillations in lamprey neurons active during locomotion. Neurosci. Lett. 60, 289–294 10.1016/0304-3940(85)90592-0 2415879

[B59] Grillner S. Zangger P. (1974). Locomotor movements generated by the deafferented spinal cord. Acta Physiol. Scand. 91, 38–39A 10.1111/j.1748-1716.1976.tb10265.x 134622

[B60] Grob M. Guertin P. A. (2007). [Role of Ca(2+) in the pacemaker-like property of spinal motoneurons]. Med. Sci. (Paris) 23, 64–66 10.1051/medsci/200723164 17212933

[B61] Guertin P. Angel M. J. Perreault M.-C. McCrea D. A. (1995). Ankle extensor group I afferents excite extensors throughout the hindlimb during fictive locomotion in the cat. J. Physiol. 487, 197–209 7473249 10.1113/jphysiol.1995.sp020871 PMC1156609

[B62] Guertin P. A. (2004). Role of NMDA receptor activation in serotonin agonist-induced air-stepping in paraplegic mice. Spinal Cord 42, 185–190 10.1038/sj.sc.3101580 14758350

[B63] Guertin P. A. (2005). Semiquantitative assessment of hindlimb movement recovery without intervention in adult paraplegic mice. Spinal Cord 43, 162–166 10.1038/sj.sc.3101701 15570318

[B64] Guertin P. A. (2008). A technological platform to optimize combinatorial treatment design and discovery for chronic spinal cord injury. J. Neurosci. Res. 86, 3039–3051 10.1002/jnr.21761 18615646

[B65] Guertin P. A. (2009a). Recovery of locomotor function with combinatory drug treatments designed to synergistically activate specific neuronal networks. Curr. Med. Chem. 16, 1366–1371 10.2174/092986709787846541 19355892

[B66] Guertin P. A. (2009b). The mammalian central pattern generator for locomotion. Brain Res. Rev. 62, 45–56 10.1016/j.brainresrev.2009.08.002 19720083

[B67] Guertin P. A. (2013). “The spinal cord: functional organization, diseases and dysfunctions,” in Neuromethods; Animal models of Spinal Cord Repair (Humana Press) (in press).

[B68] Guertin P. A. Hounsgaard J. (1998a). Chemical and electrical stimulation induce rhythmic motor activity in an in vitro preparation of the spinal cord from adult turtles. Neurosci. Lett. 245, 5–8 10.1016/S0304-3940(98)00164-5 9596342

[B69] Guertin P. A. Hounsgaard J. (1998b). NMDA-Induced intrinsic voltage oscillations depend on l-type calcium channels in spinal motoneurons of adult turtles. J. Neurophysiol. 80, 3380–3382 9862937 10.1152/jn.1998.80.6.3380

[B70] Guertin P. A. Steuer I. (2009). Key central pattern generators of the spinal cord. Neurosci. Res. J. 87, 2399–2405 10.1002/jnr.22067 19326451

[B71] Guertin P. A. Ung R. V. Rouleau P. (2010). Oral administration of a tri-therapy for central pattern generator activation in paraplegic mice: proof-of-concept of efficacy. Biotechnol. J. 5, 421–426 10.1002/biot.200900278 20349462

[B72] Guertin P. A. Ung R. V. Rouleau P. Steuer I. (2011). Effects on locomotion, muscle, bone, and blood induced by a combination therapy eliciting weight-bearing stepping in nonassisted spinal cord-transected mice. Neurorehabil. Neural Repair 25, 234–242 10.1177/1545968310378753 20952632

[B73] Gurfinkel V. S. Shik M. L. (1973). “The control of posture and locomotion,” in Motor Control, ed Gydikov Tankov Kosarov (New York: Plenum Press), 217–234

[B74] Harris-Warrick R. M. (2002). Voltage-sensitive ion channels in rhythmic motor systems. Curr. Opin. Neurobiol. 2, 646–51 [Review]. 10.1016/S0959-4388(02)00377-X 12490254

[B75] Harris-Warrick R. M. (2011). Neuromodulation and flexibility in central pattern generator networks. Curr. Opin. Neurobiol. 21, 685–692 10.1016/j.conb.2011.05.011 21646013 PMC3171584

[B76] Henneman E. (1974). “Spinal reflexes and the control of movement,” in Medical Physiology, 13th Edn, Vol. 1, ed. Mountcastle V. B. (St Louis: Mosby).

[B77] Hochman S. Gozal E. A. Hayes H. B. Anderson J. T. DeWeerth S. P. Chang Y. H. (2012). Enabling techniques for in vitro studies on mammalian spinal locomotor mechanisms. Front. Biosci. 17, 2158–2180 [Review]. 10.2741/4043 22652770 PMC7001871

[B78] Hochman S. Jordan L. M. MacDonald J. F. (1994). N-methyl-d-aspartate receptor-mediated voltage oscillations in neurons surrounding the central canal in slices of rat spinal cord. J. Neurophysiol. 72, 565–577 7983519 10.1152/jn.1994.72.2.565

[B79] Holinski B. J. Mazurek K. A. Everaert D. G. Stein R. B. Mushahwar V. K. (2011). Restoring stepping after spinal cord injury using intraspinal microstimulation and novel control strategies. Conf. Proc. IEEE Eng. Med. Biol. Soc. 2011, 5798–5801 22255658 10.1109/IEMBS.2011.6091435 PMC3276678

[B80] Holmes G. (1915). Spinal injuries of warfare. Br. Med. J. 2, 815–821 10.1136/bmj.2.2866.815 20767922 PMC2303482

[B81] Hopper S. L. DiCaprio R. A. (2004). Crustacean motor pattern generator networks. Invertebrate neural networks. Neurosignals 13, 50–69 10.1159/000076158 15004425

[B82] Hugues G. M. Wiersma C. A. G. (1960). The coordination of swimmeret movements in the crayfish, Procambarus clarkii (Girard). J. Exp. Biol. 39, 657–670

[B83] Jankowska E. (2008). Spinal interneuronal networks in the cat: elementary components. Brain Res. Rev. 57, 46–55 10.1016/j.brainresrev.2007.06.022 17884173 PMC2683333

[B84] Jankowska E. Jukes M. G. Lund S. Lundberg A. (1967a). The effect of DOPA on the spinal cord. 5. Reciprocal organization of pathways transmitting excitatory action to alpha motoneurones of flexors and extensors. Acta Physiol. Scand. 70, 369–388 10.1111/j.1748-1716.1967.tb03637.x 4293473

[B85] Jankowska E. Jukes M. G. Lund S. Lundberg A. (1967b). The effect of DOPA on the spinal cord. 6. Half-centre organization of interneurones transmitting effects from the flexor reflex afferents. Acta Physiol. Scand. 70, 389–402 10.1111/j.1748-1716.1967.tb03637.x 4294400

[B86] Landry E. S. Lapointe N. P. Rouillard C. Levesque D. Hedlund P. B. Guertin P. A. (2006a). Contribution of spinal 5-HT1A and 5-HT7 receptors to locomotor-like movement induced by 8-OH-DPAT in spinal cord-transected mice. Eur. J. Neurosci. 24, 535–546 10.1111/j.1460-9568.2006.04917.x 16836640

[B87] Landry E. S. Rouillard C. Levesque D. Guertin P. A. (2006b). Profile of immediate early gene expression in the lumbar spinal cord of low-thoracic paraplegic mice. Behav. Neurosci. 120, 1384–1388 10.1037/0735-7044.120.6.1384 17201484

[B88] Johnston M. V. Mullaney B. Blue M. E. (2003). Neurobiology of Rett syndrome. J. Child Neurol. 18, 688–692 10.1177/08830738030180100501 14649550

[B89] Jones R. Cavanna A. E. (2012). The neurobiology and treatment of restless legs syndrome. Behav. Neurol. 22713426 10.3233/BEN-2012-120271 PMC5214985

[B90] Jordan L. M. Liu J. Hedlund P. B. Akay T. Pearson K. G. (2008). Descending command systems for the initiation of locomotion in mammals. Brain Res. Rev. 57, 183–191 10.1016/j.brainresrev.2007.07.019 17928060

[B91] Juvin L. Le Gal J. P. Simmers J. Morin D. (2012). Cervicolumbar coordination in mammalian quadrupedal locomotion: role of spinal thoracic circuitry and limb sensory inputs. J. Neurosci. 32, 953–965 10.1523/JNEUROSCI.4640-11.2012 22262893 PMC6621141

[B92] Kandel E. R. Schwartz J. H. Jessell T. M. (2000). Principles of Neural Science, 4th Edn New York: McGraw-Hill

[B93] Kjaerulff O. Barajon I. Kiehn O. (1994). Sulphorhodamine-labelled cells in the neonatal rat spinal cord following chemically induced locomotor activity in vitro. J. Physiol. 478, 265–273 7525942 10.1113/jphysiol.1994.sp020248 PMC1155684

[B94] Knikou M. Angeli C. A. Ferreira C. K. Harkema S. J. (2009). Flexion reflex modulation during stepping in human spinal cord injury. Exp. Brain Res. 196, 341–351 10.1007/s00221-009-1854-x 19468720

[B95] Kristan W. B. Weeks J. C. (1983). “Neurons controlling the initiation, generation and modulation of leech swimming,” in Neural Origin of Rhythmic Movements, eds Roberts A. Roberts B. (Cambridge: Cambridge University Press), 243–260 6679114

[B96] Kuhn R. A. (1950). Functional capacity of the isolated human spinal cord. Brain 73, 1–51 10.1093/brain/73.1.1 15420313

[B97] Kullander K. Butt S. J. Lebret J. M. Lundfald L. Restrepo C. E. Rydstrom A. (2003). Role of EphA4 and EphrinB3 in local neuronal circuits that control walking. Science 299, 1889–1892 10.1126/science.1079641 12649481

[B98] Lafreniere-Roula M. McCrea D. A. (2005). Deletions of rhythmic motoneuron activity during fictive locomotion and scratch provide clues to the organization of the mammalian central pattern generator. J. Neurophysiol. 94, 1120–1132 10.1152/jn.00216.2005 15872066

[B99] Landry E. S. Guertin P. A. (2004). Differential effects of 5-HT1 and 5-HT2 receptor agonists on hindlimb movements in paraplegic mice. Prog. Neuropsychopharmacol. Biol. Psychiatry 28, 1053–1060 10.1016/j.pnpbp.2004.05.001 15380867

[B100] Langlet C. Leblond H. Rossignol S. (2005). Mid-lumbar segments are needed for the expression of locomotion in chronic spinal cats. J. Neurophysiol. 93, 2474–2488 10.1152/jn.00909.2004 15647400

[B101] Lanuza G. M. Gosgnach S. Pierani A. Jessell T. M. Goulding M. (2004). Genetic identification of spinal interneurons that coordinate left-right locomotor activity necessary for walking movements. Neuron 42, 375–386 10.1016/S0896-6273(04)00249-1 15134635

[B102] Lapointe N. P. Guertin P. A. (2008). Synergistic effects of D1/5 and 5-HT1A/7 receptor agonists on locomotor movement induction in complete spinal cord-transected mice. J. Neurophysiol. 100, 160–168 10.1152/jn.90339.2008 18480366

[B103] Lapointe N. P. Rouleau P. Ung R. V. Guertin P. A. (2009). Specific role of D1 receptors in spinal network activation and rhythmic movement induction in vertebrates. J. Physiol. 587, 1499–1511 10.1113/jphysiol.2008.166314 19204052 PMC2678221

[B104] Lapointe N. P. Ung R. V. Rouleau P. Guertin P. A. (2008). Effects of spinal alpha(2)-adrenoceptor and I(1)-imidazoline receptor activation on hindlimb movement induction in spinal cord-injured mice. J. Pharmacol. Exp. Ther. 325, 994–1006 10.1124/jpet.107.134874 18364473

[B105] Le Ray D. Juvin L. Ryczko D. Dubuc R. (2011). Chapter 4 – supraspinal control of locomotion: the mesencephalic locomotor region. Prog. Brain Res. 188, 51–70 10.1016/B978-0-444-53825-3.00009-7 21333802

[B106] Lev-Tov A. Etlin A. Blivis D. (2010). Sensory-induced activation of pattern generators in the absence of supraspinal control. Ann. N. Y. Acad. Sci. 1198, 54–62 10.1111/j.1749-6632.2009.05424.x 20536920

[B107] Lhermitte J. (1919). La section totale de la Moelle Dorsale. Bourges: Tardy Pigelet

[B108] Li X. L. Zhang W. Zhou X. Wang X. Y. Zhang H. T. Qin D. X. (2007). Temporal changes in the expression of some neurotrophins in spinal cord transected adult rats. Neuropeptides 41, 135–143 10.1016/j.npep.2007.02.001 17459471

[B109] Liu Y. C. Bailey I. Hale M. E. (2012). Alternative startle motor patterns and behaviors in the larval zebrafish (Danio rerio). J. Comp. Physiol. A Neuroethol. Sens. Neural Behav. Physiol. 198, 11–24 10.1007/s00359-011-0682-1 21983742

[B110] Lovely R. G. Gregor R. J. Roy R. R. Edgerton V. R. (1986). Effects of training on the recovery of full-weight-bearing stepping in the adult spinal cat. Exp. Neurol. 92, 421–435 10.1016/0014-4886(86)90094-4 3956672

[B111] Lukacova N. Kolesarova M. Kucharova K. Pavel J. Kolesar D. Radonak J. (2006). The effect of a spinal cord hemisection on changes in nitric oxide synthase pools in the site of injury and in regions located far away from the injured site. Cell. Mol. Neurobiol. 26, 1365–1383 10.1007/s10571-006-9092-2 PMC11520756 16786429

[B112] Lundfald L. Restrepo C. E. Butt S. J. Peng C. Y. Droho S. Endo T. (2007). Phenotype of V2-derived interneurons and their relationship to the axon guidance molecule EphA4 in the developing mouse spinal cord. Eur. J. Neurosci. 26, 2989–3002 10.1111/j.1460-9568.2007.05906.x 18028107

[B113] Ma Z. Liu T. Li X. Zhou T. Xiao L. Que H. (2006). Identification of up-regulated genes after complete spinal cord transection in adult rats. Cell. Mol. Neurobiol. 26, 277–288 10.1007/s10571-006-9046-8 16767513 PMC11520604

[B114] MacLean J. N. Schmidt B. J. Hochman S. (1997). NMDA receptor activation triggers voltage oscillations, plateau potentials and bursting in neonatal rat lumbar motoneurons in vitro. Eur. J. Neurosci. 9, 2702–2711 10.1111/j.1460-9568.1997.tb01699.x 9517475

[B115] Matsumoto N. Yoshida M. Uematsu K. (2007). Effects of partial ablation of the cerebellum on sustained swimming in goldfish. Brain Behav. Evol. 70, 105–114 10.1159/000102972 17519524

[B116] Matthews P. B. C. (1972). Mammalian Muscle Receptors and their Central Actions. Baltimore: Williams and Wilkins

[B117] Matthews P. B. C. (1991). The human stretch reflex and the motor cortex. Trends Neurosci. 14, 87–90 10.1016/0166-2236(91)90064-2 1709536

[B118] McCrea D. A. Rybak I. A. (2008). Organization of mammalian locomotor rhythm and pattern generation. Brain Res. Rev. 57, 134–146 [Review]. 10.1016/j.brainresrev.2007.08.006 17936363 PMC2214837

[B119] Molinari M. Petrosini L. (1993). Hemicerebellectomy and motor behaviour in rats. III. Kinematics of recovered spontaneous locomotion after lesions at different developmental stages. Behav. Brain Res. 54, 43–55 10.1016/0166-4328(93)90047-T 8504011

[B120] Morrison J. Birder L. Craggs M. (2005). “Neural control,” in Incontinence, eds Abrams P. Cardozo L. Khoury S. Wein A. (Jersey: Health Publications Ltd), 363–422

[B121] Moshonkina T. R. Makarovski A. N. Bogacheva I. N. Scherbakova N. A. Savohin A. A. Gerasimenko Y. P. (2012). Effects of spinal cord electrical stimulation in patients with vertebrospinal pathology. Bull. Exp. Biol. Med. 153, 16–20 10.1007/s10517-012-1632-9 22808483

[B122] Nadeau S. Jacquemin G. Fournier C. Lamarre Y. Rossignol S. (2010). Spontaneous motor rhythms of the back and legs in a patient with a complete spinal cord transection. Neurorehabil. Neural Repair 24, 377–383 10.1177/1545968309349945 20019383

[B123] Nadelhaft I. Vera P. L. (1995). Central nervous system neurons infected by pseudorabies virus injected into the rat urinary bladder following unilateral transection of the pelvic nerve. J. Comp. Neurol. 359, 443–456 10.1002/cne.903590307 7499540

[B124] Netter F. H. (2006). Atlas of Human Anatomy. Saunders/Elsevier. Available at: http://books.google.ca/books/about/Atlas_of_Human_Anatomy.html?id=8MiIcu5BB0sC

[B125] Nishimaru H. Takizawa H. Kudo N. (2000). 5-Hydroxytryptamine-induced locomotor rhythm in the neonatal mouse spinal cord in vitro. Neurosci. Lett. 280, 187–190 10.1016/S0304-3940(00)00805-3 10675792

[B126] Ollivier-Lanvin K. Krupka A. J. AuYong N. Miller K. Prilutsky B. I. Lemay M. A. (2011). Electrical stimulation of the sural cutaneous afferent nerve controls the amplitude and onset of the swing phase of locomotion in the spinal cat. J. Neurophysiol. 105, 2297–2308 10.1152/jn.00385.2010 21389308 PMC3094182

[B127] Orlovsky G. Shik M. (1976). Control of locomotion: a neurophysiological analysis of the cat locomotor system. Intern. Rev. Physiol. Ser. II 10, 281–317

[B128] Orlovsky G. N. Severin S. V. Shik M. L. (1966). Locomotion induced by stimulation of the mesencephalon. Dokl. Akad. Nauk. SSSR 169, 1223–1226 5967985

[B129] Patestas M. A. Gartner L. P. (eds). (2006). “Ascending sensory pathways,” in A Textbook of Neuroanatomy (Malden, MA: Black well Publishing), 118–133

[B130] Pearson K. G. (1993). Common principles of motor control in vertebrates and invertebrates. Annu. Rev. Neurosci. 16, 265–297 10.1146/annurev.ne.16.030193.001405 8460894

[B131] Pearson K. G. (1995). Proprioceptive regulation of locomotion. Curr. Opin. Neurobiol. 5, 786–791 10.1016/0959-4388(95)80107-3 8805415

[B132] Pearson K. G. Rossignol S. (1991). Fictive motor patterns in chronic spinal cats. J. Neurophysiol. 66, 1874–1887 1812222 10.1152/jn.1991.66.6.1874

[B133] Peng X. M. Zhou Z. G. Glorioso J. C. Fink D. J. Mata M. (2006). Tumor necrosis factor-alpha contributes to below-level neuropathic pain after spinal cord injury. Ann. Neurol. 59, 843–851 10.1002/ana.20855 16634039

[B134] Perreault M. C. Angel M. J. Guertin P. McCrea D. A. (1995). Effects of stimulation of hindlimb flexor group II afferents during fictive locomotion in the cat. J. Physiol. 487(Pt 1), 211–220 7473250 10.1113/jphysiol.1995.sp020872 PMC1156610

[B135] Perret C. Cabelguen J. M. (1980). Main characteristics of the hindlimb locomotor cycle in the decorticate cat with special reference to bifunctional muscles. Brain Res. 187, 333–352 10.1016/0006-8993(80)90207-3 7370734

[B136] Philippson M. (1905). L’autonomie et la centralisation dans le système nerveux des animaux [Autonomy and centralization in the animal nervous system]. Trav. Lab. Physiol. Inst. Solvay (Bruxelles) 7, 1–208

[B137] Pineau I. Lacroix S. (2007). Proinflammatory cytokine synthesis in the injured mouse spinal cord: multiphasic expression pattern and identification of the cell types involved. J. Comp. Neurol. 500, 267–285 10.1002/cne.21149 17111361

[B138] Reith C. A. Sillar K. T. (1998). A role for slow NMDA receptor-mediated, intrinsic neuronal oscillations in the control of fast fictive swimming in Xenopus laevis larvae. Eur. J. Neurosci. 10, 1329–1340 10.1046/j.1460-9568.1998.00144.x 9749787

[B139] Rémy-Néris O. Barbeau H. Daniel O. Boiteau F. Bussel B. (1999). Effects of intrathecal clonidine injection on spinal reflexes and human locomotion in incomplete paraplegic subjects. Exp. Brain Res. 129, 433–440 10.1007/s002210050910 10591914

[B140] Roberts B. L. van Rossem A. de Jager S. (1992). The influence of cerebellar lesions on the swimming performance of the trout. J. Exp. Biol. 167, 171–178 1634862 10.1242/jeb.167.1.171

[B141] Robertson R. M. Pearson K. G. (1985). Neural circuits in the flight system of the locust. J. Neurophysiol. 53, 110–128 2983035 10.1152/jn.1985.53.1.110

[B142] Rossignol S. Barrière G. Frigon A. Barthélemy D. Bouyer L. Provencher J. (2008). Plasticity of locomotor sensorimotor interactions after peripheral and/or spinal lesions. Brain Res. Rev. 57, 228–240 10.1016/j.brainresrev.2007.06.019 17822774

[B143] Rossignol S. Giroux N. Chau C. Marcoux J. Brustein E. Reader T. A. (2001). Pharmacological aids to locomotor training after spinal injury in the cat. J. Physiol. 533(Pt 1), 65–74 10.1111/j.1469-7793.2001.0065b.x 11351014 PMC2278596

[B144] Roux J. C. Dura E. Moncla A. Mancini J. Villard L. (2007). Treatment with desipramine improves breathing and survival in a mouse model for Rett syndrome. Eur. J. Neurosci. 25, 1915–1922 10.1111/j.1460-9568.2007.05466.x 17439480

[B145] Ruggiero D. A. Anwar M. Kim J. Sica A. L. Gootman N. Gootman P. A. (1997). Induction of c-fos gene expression by spinal cord transection in the rat. Brain Res. 763, 21–29 10.1016/S0006-8993(97)00356-9 9272824

[B146] Rye D. B. Trotti L. M. (2012). Restless legs syndrome and periodic leg movements of sleep. Neurol. Clin. 30, 1137–1166 10.1016/j.ncl.2012.08.004 23099132

[B147] Schmidt B. J. Hochman S. MacLean J. N. (1998). NMDA receptor-mediated oscillatory properties: potential role in rhythm generation in the mammalian spinal cord. Ann. N. Y. Acad. Sci. 860, 189–202 10.1111/j.1749-6632.1998.tb09049.x 9928312

[B148] Schmidt B. J. Jordan L. M. (2000). The role of serotonin in reflex modulation and locomotor rhythm production in the mammalian spinal cord. Brain Res. Bull. 53, 689–710 10.1016/S0361-9230(00)00402-0 11165804

[B149] Schmitt C. Miranpuri G. S. Dhodda V. K. Isaacson J. Vemuganti R. Resnick D. K. (2006). Changes in spinal cord injury-induced gene expression in rat are strain-dependent. Spine. J. 6, 113–119 10.1016/j.spinee.2006.06.208 16517380

[B150] Schomburg E. D. Petersen N. Barajon I. Hultborn H. (1998). Flexor reflex afferents reset the step cycle during fictive locomotion in the cat. Exp. Brain Res. 122, 339–350 10.1007/s002210050522 9808307

[B151] Schroder H. D. (1985). Anatomical and pathoanatomical studies on the spinal efferent systems innervating pelvic structures. 1. Organization of spinal nuclei in animals. 2. The nucleus X-pelvic motor system in man. J. Auton. Nerv. Syst. 14, 23–48 10.1016/0165-1838(85)90123-7 2413098

[B152] Schürks M. Bussfeld P. (2012). Multiple sclerosis and restless legs syndrome: a systematic review and meta-analysis. Eur. J. Neurol. 10.1111/j.1468-1331.2012.03873.x 23078359

[B153] Sherrington C. S. (1910). Flexion-reflex of the limb, crossed extension-reflex, and reflex stepping and standing. J. Physiol. 40, 28–121 16993027 10.1113/jphysiol.1910.sp001362 PMC1533734

[B154] Shneyder N. Harris M. K. Minagar A. (2011). Movement disorders in patients with multiple sclerosis. Handb. Clin. Neurol. 100, 307–314 10.1016/B978-0-444-52014-2.00023-9 21496590

[B155] Sigvardt K. A. Grillner S. Wallen P. Van Dongen P. A. (1985). Activation of NMDA receptors elicits fictive locomotion and bistable membrane properties in the lamprey spinal cord. Brain Res. 336, 390–395 10.1016/0006-8993(85)90676-6 2988706

[B156] Sławi၄ska U. Rossignol S. Bennett D. J. Schmidt B. J. Frigon A. Fouad K. (2012). Comment on “Restoring voluntary control of locomotion after paralyzing spinal cord injury.” Science 338, 328 10.1126/science.338.6105.328-a 23087231

[B157] Stein R. B. Capaday C. (1988). The modulation of human reflexes during functional motor tasks. Trends Neurosci. 11, 328–332 10.1016/0166-2236(88)90097-5 2465639

[B158] Stokes V. P. Andersson C. Forssberg H. (1989). Rotational and translational movement features of the pelvis and thorax during adult human locomotion. J. Biomech. 22, 43–50 10.1016/0021-9290(89)90183-8 2914971

[B159] Strupp M. Thurtell M. J. Shaikh A. G. Brandt T. Zee D. S. Leigh R. J. (2011). Pharmacotherapy of vestibular and ocular motor disorders, including nystagmus. J. Neurol. 258, 1207–1222 10.1007/s00415-011-5954-8 21461686 PMC3132281

[B160] Stuart D. G. Hultborn H. (2008). Thomas Graham Brown (1882–1965), Anders Lundberg (1920-), and the neural control of stepping. Brain Res. Rev. 59, 74–95 10.1016/j.brainresrev.2008.06.001 18582502

[B161] Sugaya K. Nishijima S. Miyazato M. Ogawa Y. (2005). Central nervous control of micturition and urine storage. J. Smooth Muscle Res. 41, 117–132 10.1540/jsmr.41.117 16006745

[B162] Swartz M. H. (1998). Textbook of Physical Diagnosis: History and Examination, 3rd Edn Philadelphia: WB Saunders Co

[B163] Székely G. Czéh G. Voros G. (1969). The activity pattern of limb muscles in freely moving normal and deafferented newts. Exp. Brain Res. 9, 53–72 5808480 10.1007/BF00235451

[B164] Tan U. (2006a). A new syndrome with quadrupedal gait, primitive speech, and severe mental retardation as a live model for human evolution. Int. J. Neurosci. 116, 361–369 10.1080/00207450500455330 16484061

[B165] Tan U. (2006b). Evidence for “Uner Tan syndrome” and the evolution of the human mind. Int. J. Neurosci. 116, 763–774 10.1080/00207450500455330 16861146

[B166] Tan U. (2008). Uner Tan syndrome: review and report of four new cases. Int. J. Neurosci. 118, 211–225 10.1080/00207450701667766 18205078

[B167] Tan U. Pençe S. Yilmaz M. Ozkur A. Karaca S. Tan M. (2008). “Uner Tan syndrome” in two Turkish families in relation to devolution and emergence of Homo erectus: neurological examination, MRI, and PET scans. Int. J. Neurosci. 118, 313–336 10.1080/00207450701667766 18300005

[B168] Tassinari C. A. Cantalupo G. Högl B. Cortelli P. Tassi L. Francione S. (2009). Neuroethological approach to frontolimbic epileptic seizures and parasomnias: the same central pattern generators for the same behaviours. Rev. Neurol. 165, 762–768 10.1016/j.neurol.2009.08.002 19733874

[B169] Tassinari C. A. Rubboli G. Gardella E. Cantalupo G. Calandra-Buonaura G. Vedovello M. (2005). Central pattern generators for a common semiology in fronto-limbic seizures and in parasomnias. A neuroethologic approach. Neurol. Sci. 26(Suppl. 3), s225–s232 10.1007/s10072-005-0492-8 16331401

[B170] Thorpe A. J. Clair A. Hochman S. Clemens S. (2011). Possible sites of therapeutic action in restless legs syndrome: focus on dopamine and α2δ ligands. Eur. Neurol. 266, 18–29 10.1159/000328431 21709418

[B171] Tillakaratne N. J. de Leon R. D. Hoang T. X. Roy R. R. Edgerton V. R. Tobin A. J. (2002). Use-dependent modulation of inhibitory capacity in the feline lumbar spinal cord. J. Neurosci. 22, 3130–3143 11943816 10.1523/JNEUROSCI.22-08-03130.2002 PMC6757541

[B172] Tresch M. C. Saltiel P. Bizzi E. (1999). The construction of movement by the spinal cord. Nat. Neurosci. 2, 162–167 10.1038/5721 10195201

[B173] Truitt W. A. Coolen L. M. (2002). Identification of a potential ejaculation generator in the spinal cord. Science 297, 1566–1569 10.1126/science.1073885 12202834

[B174] Ung R. V. Landry E. S. Rouleau P. Lapointe N. P. Rouillard C. Guertin P. A. (2008). Role of spinal 5-HT2 receptor subtypes in quipzine-induced hindlimb movements after a low-thoracic spinal cord transection. Eur. J. Neurosci. 28, 2231–2242 10.1523/JNEUROSCI.3574-07.2008 19019202

[B175] Ung R. V. Lapointe N. P. Tremblay C. Larouche A. Guertin P. A. (2007). Spontaneous recovery of hindlimb movement in completely spinal cord transected mice: a comparison of assessment methods and conditions. Spinal Cord 45, 367–379 16955071 10.1038/sj.sc.3101970

[B176] van den Brand R. Heutschi J. Barraud Q. DiGiovanna J. Bartholdi K. Huerlimann M. (2012). Restoring voluntary control of locomotion after paralyzing spinal cord injury. Science 336, 1182–1185 10.1126/science.1217416 22654062

[B177] Viala D. Buser P. (1969). The effects of DOPA and 5-HTP on rhythmic efferent discharges in hind limb nerves in the rabbit. Brain Res. 12, 437–443 10.1016/0006-8993(69)90011-0 5306638

[B178] Villablanca J. R. Hovda D. A. Jackson G. F. Gayek R. (1993). Neurological and behavioral effects of a unilateral frontal cortical lesions in fetal kittens. I. Brain morphology, movement, posture, and sensorimotor tests. Behav. Brain Res. 57, 63–77 10.1016/0166-4328(93)90062-U 8292256

[B179] Visintin M. Barbeau H. (1989). The effects of body weight support on the locomotor pattern of spastic paretic patients. Can. J. Neurol. Sci. 16, 315–325 2766124 10.1017/s0317167100029152

[B180] Wainberg M. Barbeau H. Gauthier S. (1990). The effects of cyproheptadine on locomotion and on spasticity in patients with spinal cord injuries. J. Neurol. Neurosurg. Psychiatry 53, 754–763 10.1136/jnnp.53.9.754 2246657 PMC1014252

[B181] Wang D. Grillner S. Wallén P. (2006). Effects of flufenamic acid on fictive locomotion, plateau potentials, calcium channels and NMDA receptors in the lamprey spinal cord. Neuropharmacology 51, 1038–1046 10.1016/j.neuropharm.2006.06.012 16919683

[B182] Whelan P. Bonnot A. O’Donovan M. J. (2000). Properties of rhythmic activity generated by the isolated spinal cord of the neonatal mouse. J. Neurophysiol. 84, 2821–2833 11110812 10.1152/jn.2000.84.6.2821

[B183] Wilson J. M. Hartley R. Maxwell D. J. Todd A. J. Lieberam I. Kaltschmidt J. A. (2005). Conditional rhythmicity of ventral spinal interneurons defined by expression of the Hb9 homeodomain protein. J. Neurosci. 25, 5710–5719 10.1523/JNEUROSCI.1744-05.2005 15958737 PMC6724883

[B184] Yakovlev A. G. Faden A. I. (1994). Sequential expression of c-fos protooncogene, TNF-alpha, and dynorphin genes in spinal cord following experimental traumatic injury. Mol. Chem. Neuropathol. 23, 179–190 10.1007/BF02815410 7702707

[B185] Yokota T. Hirose K. Tanabe H. Tsukagoshi H. (1991). Sleep-related periodic leg movements (nocturnal myoclonus) due to spinal cord lesion. J. Neurol. Sci. 104, 13–18 10.1016/0022-510X(91)90210-X 1919596

[B186] Zhang S. X. Huang F. Gates M. White J. Holmberg E. G. (2010). Tail nerve electrical stimulation induces body weight-supported stepping in rats with spinal cord injury. J. Neurosci. Methods 187, 183–189 10.1016/j.jneumeth.2010.01.008 20079372

[B187] Zhang Y. Narayan S. Geiman E. Lanuza G. M. Velasquez T. Shanks B. (2008). V3 spinal neurons establish a robust and balanced locomotor rhythm during walking. Neuron 60, 84–96 10.1016/j.neuron.2008.09.027 18940590 PMC2753604

